# Supplementary material for: Impact of Donor Human Milk in the Preterm Very Low Birth Weight Gut Transcriptome Profile by Use of Exfoliated Intestinal Cells
Source: Nutrients. 2019 Nov 5;11(11):2677. doi: 10.3390/nu11112677 (PMC6893464; doi:10.3390/nu11112677)
Supplement: Supplementary file 1 [file nutrients-11-02677-s001.pdf]

Supplementary material

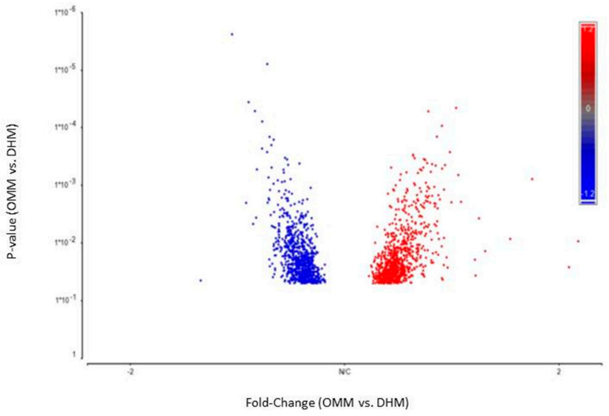

Figure S1. Volcano Plot of differentially expressed genes between preterm infants fed own mother's milk (OMM) or pasteurized donated human milk (DHM).

Table S1. The 10 most representative biological processes filtered for enrichment p-value in preterm infants.

**Biological Processes**	**p-value**	**Quantity of DEG***
Transcription, DNA-templated	$3.62x10^{-24}$	189
Regulation of transcription, DNA-templated	$5.34x10^{-22}$	188
Transport	$3.75x10^{-17}$	140
Cell cycle	$1.03x10^{-13}$	65
Gene expression	$3.38x10^{-10}$	60
Multicellular organismal development	$6.97x10^{-10}$	86

Protein transport	$1.73x10^{-09}$	56
Cell division	$2.75x10^{-09}$	39
Blood coagulation	$3.38x10^{-09}$	46
DNA repair	$8.34x10^{-09}$	39

Table S2. Differential genes in transcriptomic analysis of exfoliated epithelial intestinal cells between preterm infants fed own mother's milk (OMM) and pasteurized donated human milk (DHM).

Gene name	Gene Symbol	p-value (OMM vs. DHM)	Fold-Change (OMM vs. DHM)
Lactalbumin, alpha	LALBA	0.0024	2.92
Casein kappa	CSN3	0.0024	2.59
Casein beta	CSN2	0.0093	2.13
Cytochrome c oxidase subunit I	COX1	0.0263	2.07
Casein alpha s1	CSN1S1	0.0084	1.71
Espin	ESPN	0.0008	1.58
MTND2	ND2	0.0138	1.57
Small ubiquitin-like modifier 3	SUMO3	0.0037	1.54
Eukaryotic translation elongation factor 1 alpha 1	EEF1A1	0.0365	1.53
Ribosomal protein L10	RPL10	0.0195	1.52
Keratin associated protein 2-4	KRTAP2-4	0.0019	1.46
Serine peptidase inhibitor, Kunitz type 1	SPINT1	0.0007	1.44
Zinc finger family member 788	ZNF788	0.0000	1.43
Mitochondrial ribosomal protein L38	MRPL38	0.0020	1.41
Diacylglycerol O-acyltransferase 1	DGAT1	0.0003	1.41
Tumor protein, translationally-controlled 1	TPT1	0.0267	1.39
Retinol binding protein 2, cellular	RBP2	0.0178	1.39
Transmembrane protein 121	TMEM121	0.0004	1.38
Ankyrin repeat domain 9	ANKRD9	0.0012	1.38
Ribosomal protein L12	RPL12	0.0082	1.38
Family with sequence similarity 47, member B	FAM47B	0.0241	1.38
Acetylcholinesterase (Yt blood group)	ACHE	0.0431	1.37

Ribosomal protein S28	RPS28	0.0005	1.37
Keratin associated protein 17-1	KRTAP17-1	0.0406	1.37
Transcript Identified by aceview, Entrez Gene ID(s)	NFRKB	0.0001	1.37
Memczak2013 ANTISENSE, CDS, coding, INTERNAL best transcr	PPP6R1	0.0114	1.37
Cleavage and polyadenylation specific factor 4	CPSF4	0.0018	1.36
Ribosomal protein L36a	RPL36A	0.0085	1.36
PYD and CARD domain containing	PYCARD	0.0015	1.35
Tweety family member 1	TTYH1	0.0160	1.35
FLT3-interacting zinc finger 1	FIZ1	0.0068	1.35
Cyclin-dependent kinase 4	CDK4	0.0025	1.35
Endo-beta-N-acetylglucosaminidase	ENGASE	0.0042	1.35
Protein phosphatase 1, regulatory subunit 18	PPP1R18	0.0001	1.35
Ankyrin repeat and SOCS box containing 5	ASB5	0.0051	1.34
Regulator of G-protein signaling 14	RGS14	0.0145	1.34
Tumor suppressing subtransferable candidate 4	TSSC4	0.0005	1.34
Ring finger protein 44	RNF44	0.0021	1.34
Ribosomal protein L24	RPL24	0.0023	1.34
Acetylserotonin O-methyltransferase-like	ASMTL	0.0048	1.34
Acetylserotonin O-methyltransferase-like	ASMTL	0.0048	1.34
Fascin actin-bundling protein 2, retinal	FSCN2	0.0011	1.34
Centromere protein M	CENPM	0.0021	1.34
Zinc finger, DHHC-type containing 3	ZDHHC3	0.0218	1.33
Metallothionein 2A	MT2A	0.0152	1.33
Transmembrane protein 129, E3 ubiquitin protein ligase	TMEM129	0.0015	1.33
Cathepsin S	CTSS	0.0004	1.33
Ribosomal protein S28	RPS28	0.0088	1.32
Calcium channel, voltage-dependent, gamma subunit 7	CACNG7	0.0220	1.32
Ribosomal protein S15	RPS15	0.0199	1.32
Solute carrier family 25 (mitochondrial carrier; adenine	SLC25A6	0.0006	1.32
CD5 molecule	CD5	0.0063	1.32
6-phosphofructo-2-kinase	PFKFB4	0.0020	1.32
Cell adhesion molecule 4	CADM4	0.0011	1.31
H3 histone, family 3A	H3F3A	0.0048	1.31
Atpase, class VI, type 11A	ATP11A	0.0015	1.31
Adrenocortical dysplasia homolog	ACD	0.0001	1.31

(mouse)			
Leukotriene C4 synthase	LTC4S	0.0053	1.31
Matrix metallopeptidase 21	MMP21	0.0019	1.31
Ribosomal protein L26	RPL26	0.0133	1.31
Claudin 11	CLDN11	0.0011	1.31
Solute carrier family 25 (mitochondrial carrier; adenine nucleo	SLC25A6	0.0016	1.31
Vesicle transport through interaction with t-snares 1B	VTI1B	0.0004	1.31
Peroxisome proliferator-activated receptor alpha	PPARA	0.0013	1.30
Fizzy	FZR1	0.0180	1.30
Osteosarcoma amplified 9, endoplasmic reticulum lectin	OS9	0.0069	1.30
Structural maintenance of chromosomes 6	SMC6	0.0019	1.30
Low density lipoprotein receptor class A domain containing 3	LDLRAD3	0.0051	1.30
PIGB opposite strand 1	PIGBOS1	0.0009	1.30
Microtubule associated monooxygenase, calponin and LIM domain	MICAL2	0.0015	1.30
GLE1 RNA export mediator	GLE1	0.0249	1.30
Suppressor of cytokine signaling 7	SOCS7	0.0004	1.30
S100 calcium binding protein A8	S100A8	0.0075	1.30
Coiled-coil domain containing 130	CCDC130	0.0133	1.30
Interleukin 11 receptor, alpha	IL11RA	0.0044	1.29
Memczak2013 ANTISENSE, CDS, coding, INTERNAL best transcri	SH2B2	0.0382	1.29
Neuromedin U receptor 1	NMUR1	0.0025	1.29
Solute carrier family 26 (anion exchanger), member 1	SLC26A1	0.0020	1.29
Chromosome 11 open reading frame 96	C11orf96	0.0054	1.29
Gastrin	GAST	0.0004	1.29
Heat shock 70kda protein 8	HSPA8	0.0349	1.29
Jeck2013 ANTISENSE, coding, INTERNAL, intronic best trans	MICAL1	0.0259	1.29
Phosphatidylinositol-4-phosphate 5-kinase, type I, gamma	PIP5K1C	0.0005	1.29
Keratin 38, type I	KRT38	0.0301	1.29
Ribosomal protein S14	RPS14	0.0355	1.29
Solute carrier family 16, member 11	SLC16A11	0.0114	1.29
Small arfgap2	SMAP2	0.0023	1.29
Calcium-sensing receptor	CASR	0.0035	1.28
Metallothionein 1E	MT1E	0.0342	1.28

Brain protein I3	BRI3	0.0124	1.28
Ribosomal protein, large, P1	RPLP1	0.0064	1.28
Calcium	CAMK1G	0.0046	1.28
Ring finger protein 31	RNF31	0.0006	1.28
Solute carrier family 11 (proton-coupled divalent metal ion tra	SLC11A1	0.0235	1.28
Dihydrouridine synthase 3-like	DUS3L	0.0013	1.28
Neuroglobin	NGB	0.0047	1.28
Chromosome 1 open reading frame 145	C1orf145	0.0022	1.28
NAC alpha domain containing	NACAD	0.0168	1.28
Bone morphogenetic protein 1	BMP1	0.0069	1.28
Patched 2	PTCH2	0.0180	1.28
Transmembrane protein 238	TMEM238	0.0005	1.28
Double homeobox 1	DUX1	0.0034	1.28
Lens intrinsic membrane protein 2	LIM2	0.0247	1.28
Solute carrier family 39 (zinc transporter), member 10	SLC39A10	0.0011	1.27
Rho	ARHGEF18	0.0107	1.27
SID1 transmembrane family, member 2	SIDT2	0.0035	1.27
Protease, serine, 56	PRSS56	0.0065	1.27
L antigen family, member 3	LAGE3	0.0029	1.27
Colipase, pancreatic	CLPS	0.0108	1.27
G-2 and S-phase expressed 1	GTSE1	0.0019	1.27
Retinol binding protein 1, cellular	RBP1	0.0131	1.27
Chromosome 9 open reading frame 50	C9orf50	0.0141	1.27
Memczak2013 ANTISENSE, CDS, coding, INTERNAL best transcript	GRN	0.0135	1.27
Major histocompatibility complex, class I, L (pseudogene)	HLA-L	0.0121	1.27
KIAA0895-like	KIAA0895L	0.0006	1.27
Inositol polyphosphate-5-phosphatase D	INPP5D	0.0018	1.27
Transketolase-like 1	TKTL1	0.0158	1.27
Proline-rich transmembrane protein 2	PRRT2	0.0018	1.27
Cytochrome c oxidase subunit IV isoform 2 (lung)	COX4I2	0.0026	1.26
Ribonuclease P	RPP21	0.0107	1.26
Kruppel-like factor 1 (erythroid)	KLF1	0.0015	1.26
Enhancer of mrna decapping 4	EDC4	0.0200	1.26
Ribosomal protein L15	RPL15	0.0181	1.26
Glyceraldehyde-3-phosphate dehydrogenase	GAPDH	0.0258	1.26
C-type lectin domain family 18,	CLEC18A	0.0070	1.26

member A			
SPECC1L-ADORA2A readthrough (NMD candidate)	SPECC1L-ADORA2A	0.0145	1.26
Basic transcription factor 3	BTF3	0.0125	1.26
Transmembrane protein 132A	TMEM132A	0.0024	1.26
Hypoxia up-regulated 1	HYOU1	0.0207	1.26
Zinc finger protein 789	ZNF789	0.0024	1.26
CUB and Sushi multiple domains 1	CSMD1	0.0061	1.26
T-box 15	TBX15	0.0020	1.26
CMT1A duplicated region transcript 1	CDRT1	0.0066	1.26
Keratin 37, type I	KRT37	0.0044	1.26
Fc receptor-like B	FCRLB	0.0046	1.26
Chromosome 20 open reading frame 27	C20orf27	0.0082	1.26
Milk fat globule-EGF factor 8 protein	MFGE8	0.0010	1.26
Guanine nucleotide binding protein (G protein) alpha 12	GNA12	0.0192	1.25
Myosin, heavy chain 7B, cardiac muscle, beta	MYH7B	0.0003	1.25
N-myc downstream regulated 1	NDRG1	0.0283	1.25
Neural cell adhesion molecule 1	NCAM1	0.0269	1.25
Chromosome 7 open reading frame 73	C7orf73	0.0068	1.25
X-linked Kx blood group related 4	XKR4	0.0009	1.25
Zinc finger protein 613	ZNF613	0.0019	1.25
Ribosomal protein L4	RPL4	0.0186	1.25
Atpase, aminophospholipid transporter, class I, type 8B, memb	ATP8B3	0.0003	1.25
Acetylcholinesterase (Yt blood group)	ACHE	0.0003	1.25
CD320 molecule	CD320	0.0087	1.25
L1 cell adhesion molecule	L1CAM	0.0063	1.25
G protein-coupled receptor 6	GPR6	0.0142	1.25
Cilia and flagella associated protein 74	CFAP74	0.0076	1.25
Atpase, class V, type 10A	ATP10A	0.0114	1.25
Prosaposin	PSAP	0.0370	1.25
Left-right determination factor 2	LEFTY2	0.0181	1.25
Protease, serine 27	PRSS27	0.0139	1.24
Trna methyltransferase 2 homolog A	TRMT2A	0.0168	1.24
G-patch domain containing 8	GPATCH8	0.0078	1.24
LSM12 homolog	LSM12	0.0042	1.24
Ribosomal protein L36a	RPL36A	0.0293	1.24
Bone morphogenetic protein	BRINP2	0.0077	1.24
Memczak2013 ANTISENSE, CDS, coding, INTERNAL best transcri	RBM26	0.0296	1.24

SUZ RNA binding domain containing 1	SZRD1	0.0022	1.24
Golgin A7 family, member B	GOLGA7B	0.0035	1.24
KIAA1456	KIAA1456	0.0361	1.24
Collagen, type VIII, alpha 1	COL8A1	0.0487	1.24
Claudin 7	CLDN7	0.0056	1.24
Transcript Identified by aceview, Entrez Gene ID(s)	DNAI1	0.0067	1.24
Angiopoietin like 6	ANGPTL6	0.0232	1.24
Butyrophilin, subfamily 2, member A2	BTN2A2	0.0115	1.24
Importin 5	IPO5	0.0113	1.24
Coiled-coil domain containing 71	CCDC71	0.0087	1.24
RNA binding motif protein 38	RBM38	0.0070	1.24
Family with sequence similarity 222, member B	FAM222B	0.0227	1.24
Ankyrin repeat and SOCS box containing 8	ASB8	0.0101	1.24
Copine VII	CPNE7	0.0484	1.24
Atpase type 13A1	ATP13A1	0.0130	1.24
Synaptophysin	SYP	0.0150	1.24
Smoothened, frizzled class receptor	SMO	0.0166	1.24
Ribosomal protein S17	RPS17	0.0383	1.24
Ubia prenyltransferase domain containing 1	UBIAD1	0.0386	1.23
Carnosine synthase 1	CARNS1	0.0262	1.23
Cathepsin W	CTSW	0.0276	1.23
Janus kinase 1	JAK1	0.0317	1.23
Tumor protein p63 regulated 1	TPRG1	0.0023	1.23
Calcium channel, voltage-dependent, N type, alpha 1B subunit	CACNA1B	0.0125	1.23
Zinc finger protein 600	ZNF600	0.0134	1.23
Acid sensing ion channel family member 4	ASIC4	0.0147	1.23
Transcription factor binding to IGHM enhancer 3	TFE3	0.0224	1.23
Ring finger protein 126	RNF126	0.0154	1.23
Tubulin, beta 2B class iib	TUBB2B	0.0060	1.23
Lipin 1	LPIN1	0.0032	1.23
POM121 transmembrane nucleoporin-like 12	POM121L12	0.0053	1.23
Cramped chromatin regulator homolog 1	CRAMP1	0.0078	1.23
Jun B proto-oncogene	JUNB	0.0274	1.23
Uncharacterized LOC388282	LOC388282	0.0397	1.23
Allograft inflammatory factor 1-like	AIF1L	0.0317	1.23
Tumor necrosis factor receptor	TNFRSF25	0.0290	1.23

superfamily, member 25			
G protein-coupled receptor kinase interacting arfgap 1	GIT1	0.0278	1.23
Dynein, axonemal, assembly factor 3	DNAAF3	0.0131	1.23
BCL2-like 14 (apoptosis facilitator)	BCL2L14	0.0294	1.23
WD repeat domain 86	WDR86	0.0080	1.23
Secretoglobin, family 3A, member 1	SCGB3A1	0.0090	1.23
Zinc finger protein 749	ZNF749	0.0058	1.23
Transmembrane protein 210	TMEM210	0.0047	1.23
Serpin peptidase inhibitor, clade F (alpha-2 antiplasmin, pigm	SERPINF1	0.0071	1.23
Zhang2013 ALT_ACCEPTOR, ALT_DONOR, coding, INTERNAL, int	ZNF354B	0.0265	1.23
Insulin receptor substrate 2	IRS2	0.0012	1.23
Family with sequence similarity 109, member A	FAM109A	0.0084	1.22
Methylthioribose-1-phosphate isomerase 1	MRI1	0.0273	1.22
Dipeptidyl-peptidase 3	DPP3	0.0012	1.22
Suppressor of IKBKE 1	SIKE1	0.0202	1.22
Vascular endothelial growth factor B	VEGFB	0.0260	1.22
Cathepsin V	CTSV	0.0121	1.22
Rabphilin 3A-like (without C2 domains)	RPH3AL	0.0104	1.22
Jeck2013 ANTISENSE, CDS, coding, INTERNAL, intronic, OVCODE	MYH9	0.0176	1.22
SH3 and multiple ankyrin repeat domains 2	SHANK2	0.0252	1.22
Regulatory factor X, 1 (influences HLA class II expression)	RFX1	0.0041	1.22
Microrna 1199	MIR1199	0.0140	1.22
Chemokine (C-X-C motif) ligand 1 (melanoma growth stimulating act	CXCL1	0.0162	1.22
Potassium channel, voltage gated eag related subfamily H, memb	KCNH6	0.0161	1.22
Ras homolog family member J	RHOJ	0.0067	1.22
ALDH1L1 antisense RNA 1	ALDH1L1-AS1	0.0361	1.22
Cyclin-dependent kinase 6	CDK6	0.0018	1.22
Oxysterol binding protein-like 2	OSBPL2	0.0006	1.22
CD63 molecule	CD63	0.0445	1.22
G protein-coupled receptor 39	GPR39	0.0096	1.22
Polymerase (DNA directed), lambda	POLL	0.0013	1.22
Sphingomyelin phosphodiesterase 4, neutral membrane (neutral s	SMPD4	0.0415	1.22
Kelch-like family member 29	KLHL29	0.0030	1.22
Arginine vasopressin receptor 1A	AVPR1A	0.0131	1.22

CBP80	CTIF	0.0060	1.22
ABO blood group (transferase A, alpha 1-3-N-acetylgalactosaminyltra	ABO	0.0102	1.22
Transmembrane protein 160	TMEM160	0.0365	1.22
LIM and calponin homology domains 1	LIMCH1	0.0308	1.22
GINS complex subunit 3 (Psf3 homolog)	GINS3	0.0128	1.22
Late endosomal	LAMTOR4	0.0120	1.22
Ring finger protein 223	RNF223	0.0137	1.22
LY6	LYPD6	0.0067	1.22
Retinoic acid receptor responder (tazarotene induced) 2	RARRES2	0.0059	1.22
Phenylethanolamine N-methyltransferase	PNMT	0.0022	1.22
Olfactory receptor, family 6, subfamily Q, member 1 (gene	OR6Q1	0.0135	1.22
Family with sequence similarity 131, member A	FAM131A	0.0098	1.22
Natural killer cell granule protein 7	NKG7	0.0186	1.22
RAD9 checkpoint clamp component A	RAD9A	0.0404	1.21
Transient receptor potential cation channel, subfamily M, member	TRPM2	0.0103	1.21
ARPC4-TTLL3 readthrough	ARPC4-TTLL3	0.0096	1.21
Protein kinase domain containing, cytoplasmic	PKDCC	0.0289	1.21
Cytochrome c oxidase subunit viia polypeptide 1 (muscle)	COX7A1	0.0266	1.21
Latent transforming growth factor beta binding protein 2	LTBP2	0.0344	1.21
Carbonic anhydrase XI	CA11	0.0327	1.21
Chromosome 17 open reading frame 58	C17orf58	0.0025	1.21
Transmembrane protein 55A	TMEM55A	0.0167	1.21
Lin-7 homolog C (C. Elegans)	LIN7C	0.0121	1.21
Chromosome 9 open reading frame 62	C9orf62	0.0016	1.21
PDZ domain containing 4	PDZD4	0.0018	1.21
Mitogen-activated protein kinase kinase 7	MAP2K7	0.0089	1.21
Serpin peptidase inhibitor, clade F (alpha-2 antiplasmin, p	SERPINF2	0.0100	1.21
Cold inducible RNA binding protein	CIRBP	0.0405	1.21
Thyroid peroxidase	TPO	0.0346	1.21
Ribosomal protein S19 binding protein 1	RPS19BP1	0.0454	1.21

Undifferentiated embryonic cell transcription factor 1	UTF1	0.0197	1.21
Double homeobox 3	DUX3	0.0028	1.21
Neurotrophin 4	NTF4	0.0079	1.21
Cystatin B (stefin B)	CSTB	0.0248	1.21
Ribosomal protein L6	RPL6	0.0053	1.21
Transcription elongation factor B polypeptide 3C-like	TCEB3CL	0.0363	1.21
C-terminal binding protein 1	CTBP1	0.0089	1.21
Transmembrane 4 L six family member 5	TM4SF5	0.0163	1.21
Autophagy related 16-like 2	ATG16L2	0.0246	1.21
Transport and golgi organization 6 homolog	TANGO6	0.0295	1.21
Hes family bhlh transcription factor 7	HES7	0.0443	1.21
Chromosome 22 open reading frame 29	C22orf29	0.0105	1.21
V-set and immunoglobulin domain containing 8	VSIG8	0.0324	1.21
Vasorin	VASN	0.0319	1.21
Gasdermin C	GSDMC	0.0360	1.21
Chromosome 9 open reading frame 170	C9orf170	0.0092	1.21
G protein-coupled receptor 146	GPR146	0.0284	1.21
Ubiquitin-like with PHD and ring finger domains 2, E3 ubiquitin p	UHRF2	0.0128	1.21
Zinc finger protein 585B	ZNF585B	0.0262	1.21
Proline rich 23A	PRR23A	0.0350	1.21
Integrator complex subunit 5	INTS5	0.0026	1.21
Synaptosome associated protein 47kda	SNAP47	0.0229	1.21
S100 calcium binding protein A3	S100A3	0.0062	1.21
Ubiquitin associated protein 1 like	UBAP1L	0.0212	1.21
PR domain containing 10	PRDM10	0.0095	1.21
Tumor suppressor candidate 1	TUSC1	0.0095	1.21
EP300 interacting inhibitor of differentiation 2	EID2	0.0059	1.20
Cytochrome P450, family 26, subfamily C, polypeptide 1	CYP26C1	0.0106	1.20
Proline dehydrogenase (oxidase) 1	PRODH	0.0204	1.20
NADH dehydrogenase (ubiquinone) complex I, assembly factor 3	NDUFAF3	0.0312	1.20
TM4SF19-TCTEX1D2 readthrough (NMD candidate)	TM4SF19-TCTEX1D2	0.0020	1.20
Pleckstrin 2	PLEK2	0.0019	1.20
Jeck2013 ANTISENSE, CDS, coding,	MEN1	0.0196	1.20

INTERNAL, OVCODE, OVEXON b			
HECT domain containing E3 ubiquitin protein ligase 3	HECTD3	0.0346	1.20
Translocase of outer mitochondrial membrane 7 homolog (yeast)	TOMM7	0.0284	1.20
Activating transcription factor 5	ATF5	0.0361	1.20
Phosphoenolpyruvate carboxykinase 2 (mitochondrial)	PCK2	0.0238	1.20
MPV17 mitochondrial membrane protein-like 2	MPV17L2	0.0327	1.20
UDP-glucose ceramide glucosyltransferase	UGCG	0.0224	1.20
SCO1 cytochrome c oxidase assembly protein	SCO1	0.0478	1.20
Long intergenic non-protein coding RNA 92	LINC00092	0.0293	1.20
HGH1 homolog	HGH1	0.0201	1.20
Transcript Identified by aceview, Entrez Gene ID(	KLHDC4	0.0258	1.20
Tripartite motif containing 68	TRIM68	0.0196	1.20
Camp responsive element modulator	CREM	0.0133	1.20
Solute carrier family 22, member 17	SLC22A17	0.0257	1.20
SYS1-DBNDD2 readthrough (NMD candidate)	SYS1-DBNDD2	0.0145	1.20
Acyl-coa thioesterase 7	ACOT7	0.0285	1.20
Tubulin, gamma 1	TUBG1	0.0057	1.20
HAUS augmin like complex subunit 3	HAUS3	0.0049	1.20
LY6	LYPD3	0.0312	1.20
PRAME family member 7	PRAMEF7	0.0055	1.20
BAH domain and coiled-coil containing 1	BAHCC1	0.0148	1.20
Ephrin-B1	EFNB1	0.0106	1.20
Ligase III, DNA, ATP-dependent	LIG3	0.0302	1.20
V-myb avian myeloblastosis viral oncogene homolog-like 2	MYBL2	0.0245	1.20
Lactate dehydrogenase D	LDHD	0.0181	1.20
Serine	SRSF4	0.0380	1.20
Adhesion regulating molecule 1	ADRM1	0.0279	1.20
Potassium channel, two pore domain subfamily K, member 5	KCNK5	0.0006	1.20
Rho	ARHGEF2	0.0286	1.20
Midnolin	MIDN	0.0479	1.20
Forkhead box C1	FOXC1	0.0121	1.20
Megakaryoblastic leukemia (translocation) 1	MKL1	0.0131	1.20
Fatty acid synthase	FASN	0.0070	1.20

Mitogen-activated protein kinase kinase kinase 3	MAP3K3	0.0338	1.20
Ankyrin repeat domain 29	ANKRD29	0.0040	1.20
Nucleolar complex associated 4 homolog	NOC4L	0.0273	1.20
MAP7 domain containing 2	MAP7D2	0.0331	1.20
Nucleobindin 1	NUCB1	0.0186	1.20
NFKB inhibitor interacting Ras-like 2	NKIRAS2	0.0382	1.20
STIP1 homology and U-box containing protein 1, E3 ubiquitin pr	STUB1	0.0358	1.20
Synaptosome associated protein 91kda	SNAP91	0.0008	1.20
Zinc finger CCCH-type containing 7B	ZC3H7B	0.0403	1.19
Rho gtpase activating protein 19	ARHGAP19	0.0223	1.19
Transcript Identified by aceview, Entrez Gene ID(s) 158219	TTC39B	0.0228	1.19
Neurensin 2	NRSN2	0.0040	1.19
Mitochondrial ribosomal protein L55	MRPL55	0.0471	1.19
C1q and tumor necrosis factor related protein 4	C1QTNF4	0.0388	1.19
NK2 homeobox 8	NKX2-8	0.0439	1.19
Wilms tumor 1 interacting protein	WTIP	0.0423	1.19
Myozenin 3	MYOZ3	0.0218	1.19
Syncoilin, intermediate filament protein	SYNC	0.0363	1.19
Aprataxin	APTX	0.0194	1.19
Mortality factor 4 like 2	MORF4L2	0.0123	1.19
Calcium	CAMKK2	0.0193	1.19
Sex comb on midleg-like 4 (Drosophila)	SCML4	0.0440	1.19
F11 antisense RNA 1	F11-AS1	0.0217	1.19
Memczak2013 ANTISENSE, CDS, coding, INTERNAL best transcri	USP9X	0.0472	1.19
Breast cancer metastasis suppressor 1	BRMS1	0.0026	1.19
Msh homeobox 1	MSX1	0.0391	1.19
IGF like family member 3	IGFL3	0.0394	1.19
Major histocompatibility complex, class II, DM alpha	HLA-DMA	0.0231	1.19
Transmembrane protein 86B	TMEM86B	0.0091	1.19
Family with sequence similarity 120A	FAM120A	0.0289	1.19
Methyltransferase like 13	METTL13	0.0297	1.19
Coiled-coil domain containing 159	CCDC159	0.0262	1.19
Thyroid hormone responsive	THRSP	0.0367	1.19
Collagen, type I, alpha 1	COL1A1	0.0215	1.19

CDK2-associated, cullin domain 1	CACUL1	0.0421	1.19
Zinc finger protein 44	ZNF44	0.0304	1.19
Chromosome 20 open reading frame 173	C20orf173	0.0019	1.19
Ribosomal protein S5	RPS5	0.0060	1.19
Nuclear receptor subfamily 2, group C, member 2	NR2C2	0.0328	1.19
N-deacetylase	NDST2	0.0191	1.19
SIX homeobox 2	SIX2	0.0220	1.19
EPH receptor B6	EPHB6	0.0141	1.19
Forkhead-associated (FHA) phosphopeptide binding domain 1	FHAD1	0.0143	1.19
EXOC3 antisense RNA 1	EXOC3-AS1	0.0094	1.19
Folylpolyglutamate synthase	FPGS	0.0123	1.19
Parkinson disease 7 domain containing 1	PDDC1	0.0219	1.19
Mitochondrial ribosomal protein S14	MRPS14	0.0017	1.19
Purinergic receptor P2X, ligand gated ion channel, 3	P2RX3	0.0117	1.19
CREB	CREBZF	0.0201	1.19
Zinc finger protein 814	ZNF814	0.0403	1.19
Junctophilin 2	JPH2	0.0496	1.19
Memczak2013 ANTISENSE, coding, INTERNAL, intronic best t	NDUFA10	0.0137	1.19
Olfactory receptor, family 6, subfamily B, member 3	OR6B3	0.0311	1.19
Protein tyrosine phosphatase, receptor type, C-associated prote	PTPRCAP	0.0118	1.19
Proliferating cell nuclear antigen	PCNA	0.0120	1.19
Lectin, galactoside-binding, soluble, 7B	LGALS7B	0.0255	1.19
Integrin alpha 4	ITGA4	0.0319	1.19
UDP-glcnac:betagal beta-1,3-N-acetylglucosaminyltransferase 6	B3GNT6	0.0206	1.19
Pleckstrin homology domain containing, family B (evectins) m	PLEKHB2	0.0179	1.19
Synthetic construct Homo sapiens clone IMAGE:100062553, MGC:1905	BAGE5	0.0401	1.19
Leucine rich repeat containing 4B	LRRC4B	0.0433	1.19
Spermatogenesis associated 2	SPATA2	0.0291	1.19
Mediator complex subunit 9	MED9	0.0384	1.19
Trans-golgi network vesicle protein 23 homolog B (S. Cerevisiae)	TVP23B	0.0212	1.18
Apolipoprotein B mrna editing enzyme, catalytic polypeptide-lik	APOBEC2	0.0453	1.18
Phospholipid phosphatase 6	PLPP6	0.0161	1.18

Apolipoprotein B mrna editing enzyme, catalytic polypeptide	APOBEC3H	0.0184	1.18
Kinesin family member 7	KIF7	0.0303	1.18
RAB26, member RAS oncogene family	RAB26	0.0439	1.18
Serine	SRSF8	0.0122	1.18
High mobility group AT-hook 2	HMGA2	0.0266	1.18
Family with sequence similarity 212, member B	FAM212B	0.0418	1.18
5-hydroxytryptamine (serotonin) receptor 1B, G protein-coupled	HTR1B	0.0292	1.18
Transmembrane protein 52B	TMEM52B	0.0265	1.18
Tumor necrosis factor receptor superfamily, member 8	TNFRSF8	0.0188	1.18
Microtubule associated serine	MAST1	0.0320	1.18
Heterogeneous nuclear ribonucleoprotein A3	HNRNPA3	0.0150	1.18
Glucocorticoid modulatory element binding protein 2	GMEB2	0.0318	1.18
Papilin, proteoglycan-like sulfated glycoprotein	PAPLN	0.0261	1.18
Mitochondrial ribosome-associated gtpase 2	MTG2	0.0443	1.18
Gliomedin	GLDN	0.0318	1.18
Phosphoribosyl pyrophosphate synthetase 2	PRPS2	0.0489	1.18
DAB2 interacting protein	DAB2IP	0.0334	1.18
Solute carrier family 52 (riboflavin transporter), member 1	SLC52A1	0.0238	1.18
Poly(A) binding protein, cytoplasmic 3	PABPC3	0.0171	1.18
Nuclear receptor interacting protein 2	NRIP2	0.0074	1.18
Serine	SRRM3	0.0351	1.18
Trophinin associated protein	TROAP	0.0484	1.18
Mastermind-like transcriptional coactivator 1	MAML1	0.0292	1.18
Gap junction protein beta 5	GJB5	0.0203	1.18
Transmembrane protein 216	TMEM216	0.0452	1.18
S-antigen; retina and pineal gland (arrestin)	SAG	0.0047	1.18
Major histocompatibility complex, class II, DQ alpha	HLA-DQA2	0.0121	1.18
Transcript Identified by aceview, Entrez Gene ID(s)	HCRP1	0.0026	1.18
RAB11 family interacting protein 5 (class I)	RAB11FIP5	0.0302	1.18

Sulfotransferase family 2B member 1	SULT2B1	0.0249	1.18
Iglon family member 5	IGLON5	0.0345	1.18
Serine dehydratase-like	SDSL	0.0296	1.18
Laminin, gamma 3	LAMC3	0.0319	1.18
IMP3, U3 small nucleolar ribonucleoprotein	IMP3	0.0075	1.18
Acyl-coa thioesterase 2	ACOT2	0.0286	1.18
Proline-rich nuclear receptor coactivator 2	PNRC2	0.0478	1.18
Inhibitor of CDK, cyclin A1 interacting protein 1	INCA1	0.0032	1.18
Cadherin 3, type 1, P-cadherin (placental)	CDH3	0.0335	1.18
Rho guanine nucleotide exchange factor 19	ARHGEF19	0.0056	1.18
Sushi domain containing 2	SUSD2	0.0327	1.18
PALM2-AKAP2 readthrough	PALM2-AKAP2	0.0455	1.18
Tetraspanin 31	TSPAN31	0.0290	1.18
Lysophosphatidic acid receptor 3	LPAR3	0.0123	1.18
Valyl-trna synthetase 2, mitochondrial	VARS2	0.0010	1.18
Transmembrane protein 189	TMEM189	0.0420	1.18
WD repeat and FYVE domain containing 1	WDFY1	0.0224	1.18
Sterol regulatory element binding transcription factor 1	SREBF1	0.0457	1.18
Phosphorylase, glycogen, muscle	PYGM	0.0239	1.18
K(lysine) acetyltransferase 6B	KAT6B	0.0160	1.18
V-set and immunoglobulin domain containing 1	VSIG1	0.0431	1.18
Ribosomal protein S6 kinase, 90kda, polypeptide 1	RPS6KA1	0.0384	1.18
Eukaryotic translation initiation factor 4H	EIF4H	0.0224	1.18
Peptidylprolyl isomerase F	PPIF	0.0145	1.18
Naked cuticle homolog 2 (Drosophila)	NKD2	0.0190	1.18
Ribosomal protein S25	RPS25	0.0194	1.18
Tenascin XB	TNXB	0.0367	1.18
Protein phosphatase 1, regulatory subunit 3D	PPP1R3D	0.0136	1.18
Insulin like growth factor binding protein-like 1	IGFBPL1	0.0134	1.18
NK1 homeobox 2	NKX1-2	0.0340	1.18
Proteasome 26S subunit, non-atpase 8	PSMD8	0.0197	1.18

Coiled-coil domain containing 64	CCDC64	0.0479	1.18
Translocase of inner mitochondrial membrane 13 homolog (yeast)	TIMM13	0.0277	1.18
Transcript Identified by aceview, Entrez Gene ID(s	CYB5D2	0.0086	1.18
Transcription elongation factor B polypeptide 3C (elongin A3)	TCEB3C	0.0445	1.18
Dehydrogenase	DHRS9	0.0039	1.18
Hepcidin antimicrobial peptide	HAMP	0.0328	1.18
KH domain containing, RNA binding, signal transduction associat	KHDRBS2	0.0320	1.18
Chromosome 3 open reading frame 84	C3orf84	0.0340	1.18
Lysine (K)-specific demethylase 5D	KDM5D	0.0160	1.18
Mucolipin 1	MCOLN1	0.0358	1.18
Kinesin light chain 2	KLC2	0.0452	1.18
Cadherin 12, type 2 (N-cadherin 2)	CDH12	0.0272	1.18
X-prolyl aminopeptidase (aminopeptidase P) 2, membrane-bound	XPNPEP2	0.0215	1.18
G protein-coupled receptor 135	GPR135	0.0078	1.18
Actin binding LIM protein family, member 2	ABLIM2	0.0323	1.18
Peroxisomal biogenesis factor 11 alpha	PEX11A	0.0274	1.18
Cell division cycle 26	CDC26	0.0241	1.17
Proline rich 25	PRR25	0.0147	1.17
Contactin associated protein 1	CNTNAP1	0.0088	1.17
Keratin associated protein 5-9	KRTAP5-9	0.0218	1.17
Ribosomal protein L23	RPL23	0.0294	1.17
Collagen, type IV, alpha 2	COL4A2	0.0217	1.17
Transcription factor AP-2 epsilon (activating enhancer binding p	TFAP2E	0.0220	1.17
NME	NME3	0.0444	1.17
Epithelial membrane protein 3	EMP3	0.0130	1.17
Gametogenetin binding protein 2	GGNBP2	0.0138	1.17
Cancer	CT83	0.0280	1.17
Potassium channel, voltage gated KQT-like subfamily Q, member 2	KCNQ2	0.0252	1.17
Transmembrane protein 43	TMEM43	0.0282	1.17
SEC22 homolog C, vesicle trafficking protein	SEC22C	0.0012	1.17
S-antigen; retina and pineal gland (arrestin)	SAG	0.0123	1.17
Polymerase (RNA) III (DNA directed) polypeptide D, 44kda	POLR3D	0.0318	1.17
Chromosome 17 open reading	C17orf67	0.0364	1.17

frame 67			
Otoferlin	OTOF	0.0106	1.17
ZNF252P antisense RNA 1	ZNF252P-AS1	0.0360	1.17
Lymphocyte antigen 6 complex, locus G6C	LY6G6C	0.0172	1.17
LIM domain only 4	LMO4	0.0489	1.17
Solute carrier family 27 (fatty acid transporter), member 6	SLC27A6	0.0068	1.17
Chloride intracellular channel 1	CLIC1	0.0193	1.17
EPM2A (laforin) interacting protein 1	EPM2AIP1	0.0175	1.17
Vitamin K epoxide reductase complex subunit 1 like 1	VKORC1L1	0.0344	1.17
Coiled-coil domain containing 17	CCDC17	0.0156	1.17
Cell division cycle associated 5	CDCA5	0.0464	1.17
Dnaj (Hsp40) homolog, subfamily B, member 1	DNAJB1	0.0390	1.17
Zinc finger protein 321, pseudogene	ZNF321P	0.0365	1.17
Phospholipase C, beta 2	PLCB2	0.0244	1.17
Rab interacting lysosomal protein-like 1	RILPL1	0.0115	1.17
Heat shock 70kda protein 14	HSPA14	0.0437	1.17
Mitochondrial ribosomal protein S26	MRPS26	0.0149	1.17
Dishevelled segment polarity protein 3	DVL3	0.0347	1.17
Roundabout guidance receptor 3	ROBO3	0.0184	1.17
Voltage-dependent anion channel 1	VDAC1	0.0206	1.17
Chemokine (C-C motif) ligand 26	CCL26	0.0264	1.17
Transmembrane protein 237	TMEM237	0.0426	1.17
Dipeptidyl-peptidase 6	DPP6	0.0088	1.17
BCL2-like 14 (apoptosis facilitator)	BCL2L14	0.0012	1.17
Tryptase gamma 1	TPSG1	0.0165	1.17
Thyrotrophic embryonic factor	TEF	0.0221	1.17
Solute carrier family 6 (neutral amino acid transporter), me	SLC6A19	0.0401	1.17
Tripartite motif containing 45	TRIM45	0.0306	1.17
Adhesion G protein-coupled receptor G3	ADGRG3	0.0179	1.17
Iroquois homeobox 2	IRX2	0.0357	1.17
Prolactin releasing hormone	PRLH	0.0388	1.17
Ewing tumor-associated antigen 1	ETAA1	0.0233	1.17
Ribosomal protein L7a	RPL7A	0.0078	1.17
CD209 molecule	CD209	0.0310	1.17
Atpase, H+ transporting, lysosomal accessory protein 1	ATP6AP1	0.0448	1.17
Chromosome X open reading frame	CXorf40A	0.0180	1.17

40A			
Family with sequence similarity 197, Y-linked, member	FAM197Y1	0.0067	1.17
Purinergic receptor P2X, ligand gated ion channel, 6	P2RX6	0.0288	1.17
Neurofibromin 2 (merlin)	NF2	0.0046	1.17
ST6 (alpha-N-acetyl-neuraminyl-2,3-beta-galactosyl-1,3)-N-ac	ST6GALNAC2	0.0319	1.17
Macrophage erythroblast attacher	MAEA	0.0295	1.17
Zinc finger CCCH-type containing 8	ZC3H8	0.0229	1.17
Double homeobox 4 like 7	DUX4L7	0.0181	1.17
Chromosome 19 open reading frame 60	C19orf60	0.0370	1.17
Solute carrier family 47 (multidrug and toxin extrusion), membe	SLC47A1	0.0226	1.17
Leucine rich repeat containing 55	LRRC55	0.0249	1.17
Cytokine receptor-like factor 2	CRLF2	0.0338	1.17
Zinc finger protein 764	ZNF764	0.0290	1.17
PRAME family member 25	PRAMEF25	0.0335	1.17
Memczak2013 ANTISENSE, CDS, coding, INTERNAL best transcript	MPO	0.0136	1.16
Glutamate decarboxylase like 1	GADL1	0.0075	1.16
CKLF-like MARVEL transmembrane domain containing 5	CMTM5	0.0134	1.16
Von Willebrand factor A domain containing 5B1	VWA5B1	0.0454	1.16
Tetratricopeptide repeat domain 34	TTC34	0.0401	1.16
Short stature homeobox	SHOX	0.0422	1.16
Chromosome 17 open reading frame 89	C17orf89	0.0232	1.16
Regulator of G-protein signaling 3	RGS3	0.0289	1.16
Hedgehog acyltransferase	HHAT	0.0094	1.16
Cytidine monophosphate (UMP-CMP) kinase 1, cytosolic	CMPK1	0.0152	1.16
Solute carrier family 7 (amino acid transporter light chain, L s	SLC7A5	0.0273	1.16
Peroxisomal biogenesis factor 12	PEX12	0.0024	1.16
Contactin associated protein-like 2	CNTNAP2	0.0497	1.16
Tyrosyl-trna synthetase 2, mitochondrial	YARS2	0.0005	1.16
Like-glycosyltransferase	LARGE	0.0211	1.16
Interferon, lambda 4 (gene	IFNL4	0.0146	1.16
N-acetyltransferase 14 (GCN5-related, putative)	NAT14	0.0186	1.16
Proline, glutamate and leucine rich protein 1	PELP1	0.0437	1.16
Zinc finger protein 547	ZNF547	0.0069	1.16

Zinc finger protein 114	ZNF114	0.0232	1.16
Small nuclear ribonucleoprotein polypeptides B and B1	SNRPB	0.0402	1.16
Stromal cell-derived factor 2-like 1	SDF2L1	0.0167	1.16
Leukemia NUP98 fusion partner 1	LNP1	0.0226	1.16
Carcinoembryonic antigen-related cell adhesion molecule 18	CEACAM18	0.0366	1.16
Inosine triphosphatase (nucleoside triphosphate pyrophosphatase	ITPA	0.0330	1.16
Solute carrier family 45, member 4	SLC45A4	0.0441	1.16
Peroxisome proliferator-activated receptor delta	PPARD	0.0218	1.16
Homo sapiens similar to testis specific protein, Y-linked 1,	LOC728395	0.0254	1.16
Family with sequence similarity 153, member A	FAM153A	0.0111	1.16
Arrestin, beta 2	ARRB2	0.0498	1.16
Rho gtpase activating protein 11B	ARHGAP11B	0.0458	1.16
Chromosome 6 open reading frame 120	C6orf120	0.0294	1.16
Peroxisomal biogenesis factor 19	PEX19	0.0156	1.16
SPT20 homolog, SAGA complex component-like 2	SUPT20HL2	0.0267	1.16
Growth factor receptor bound protein 7	GRB7	0.0303	1.16
Chromosome 14 open reading frame 119	C14orf119	0.0204	1.16
Diacylglycerol lipase, beta	DAGLB	0.0131	1.16
Mitochondrial ribosomal protein S18B	MRPS18B	0.0259	1.16
Crystallin gamma D	CRYGD	0.0198	1.16
SHC (Src homology 2 domain containing) transforming protein 2	SHC2	0.0337	1.16
Chloride channel, voltage-sensitive Ka	CLCNKA	0.0247	1.16
Olfactory marker protein	OMP	0.0310	1.16
Src-related kinase lacking C-terminal regulatory tyrosine and N-te	SRMS	0.0206	1.16
HKR1, GLI-Kruppel zinc finger family member	HKR1	0.0257	1.16
Dystrobrevin binding protein 1	DTNBP1	0.0151	1.16
Coxsackie virus and adenovirus receptor	CXADR	0.0023	1.16
Ataxin 10	ATXN10	0.0426	1.16
Chromosome 14 open reading frame 169	C14orf169	0.0099	1.16
SMAD family member 9	SMAD9	0.0048	1.16

MRGPRG antisense RNA 1	MRGPRG-AS1	0.0385	1.16
Chromosome 15 open reading frame 54	C15orf54	0.0389	1.16
Calmodulin binding transcription activator 1	CAMTA1	0.0481	1.16
SEC31 homolog B, COPII coat complex component	SEC31B	0.0099	1.16
Proline rich 22	PRR22	0.0390	1.16
Chromosome 19 open reading frame 73	C19orf73	0.0433	1.16
Zinc finger, FYVE domain containing 19	ZFYVE19	0.0284	1.15
Centrosomal protein 135kda	CEP135	0.0422	1.15
Kinesin light chain 3	KLC3	0.0339	1.15
Olfactory receptor, family 2, subfamily T, member 1	OR2T1	0.0321	1.15
Bladder cancer associated protein	BLCAP	0.0494	1.15
Coiled-coil domain containing 125	CCDC125	0.0198	1.15
Lysosomal protein transmembrane 5	LAPTM5	0.0128	1.15
Carbohydrate (N-acetylglucosamine-6-O) sulfotransferase 2	CHST2	0.0218	1.15
SAGA complex associated factor 29 pseudogene	LOC388242	0.0216	1.15
Aldo-keto reductase family 7-like (gene	AKR7L	0.0316	1.15
Desert hedgehog	DHH	0.0434	1.15
Follistatin-like 4	FSTL4	0.0416	1.15
Chromosome 10 open reading frame 82	C10orf82	0.0319	1.15
Family with sequence similarity 124 member A	FAM124A	0.0147	1.15
Zinc finger protein 780A	ZNF780A	0.0463	1.15
Calcium channel, voltage-dependent, L type, alpha 1S subunit	CACNA1S	0.0357	1.15
Transmembrane protein 63C	TMEM63C	0.0130	1.15
Family with sequence similarity 26, member F	FAM26F	0.0372	1.15
Transmembrane protein 183B	TMEM183B	0.0208	1.15
Dopamine receptor D5	DRD5	0.0319	1.15
BCL2 binding component 3	BBC3	0.0304	1.15
Apoptotic chromatin condensation inducer 1	ACIN1	0.0118	1.15
Estrogen-related receptor alpha	ESRRA	0.0287	1.15
CXXC finger protein 5	CXXC5	0.0218	1.15
Dedicator of cytokinesis 10	DOCK10	0.0071	1.15
Solute carrier family 6	SLC6A4	0.0316	1.15

(neurotransmitter transporter), member 4			
Perilipin 5	PLIN5	0.0346	1.15
Mitogen-activated protein kinase 3	MAPK3	0.0410	1.15
Solute carrier family 2 (facilitated glucose transporter), membe	SLC2A4	0.0370	1.15
Tumor necrosis factor receptor superfamily, member 10c, decoy	TNFRSF10C	0.0197	1.15
Chitinase 3-like 1 (cartilage glycoprotein-39)	CHI3L1	0.0472	1.15
Xeroderma pigmentosum, complementation group C	XPC	0.0164	1.15
Phosphoserine phosphatase	PSPH	0.0079	1.15
Interleukin 36, gamma	IL36G	0.0337	1.15
Retinoid X receptor alpha	RXRA	0.0337	1.15
Ferredoxin 1	FDX1	0.0234	1.15
Ras homolog family member Q	RHOQ	0.0014	1.15
Solute carrier family 9, subfamily A (NHE3, cation proton anti	SLC9A3R1	0.0500	1.15
Pleckstrin homology domain containing, family G (with rhogef	PLEKHG6	0.0114	1.15
Synaptogyrin 4	SYNGR4	0.0413	1.15
Par-3 family cell polarity regulator beta	PARD3B	0.0067	1.15
CWC15 spliceosome-associated protein	CWC15	0.0487	1.15
JRK-like	JRKL	0.0319	1.15
Misshapen-like kinase 1	MINK1	0.0247	1.15
LSM6 homolog, U6 small nuclear RNA and mrna degradation associated	LSM6	0.0315	1.15
Crystallin beta B2	CRYBB2	0.0391	1.15
Coatomer protein complex subunit zeta 2	COPZ2	0.0225	1.15
Karyopherin alpha 4 (importin alpha 3)	KPNA4	0.0414	1.15
Sirtuin 6	SIRT6	0.0417	1.15
Chromosome 12 open reading frame 71	C12orf71	0.0342	1.15
Steroidogenic acute regulatory protein	STAR	0.0224	1.15
NSE1 homolog, SMC5-SMC6 complex component	NSMCE1	0.0398	1.15
LEM domain containing 3	LEMD3	0.0429	1.15
KIAA1671	KIAA1671	0.0296	1.15
Absent in melanoma 1-like	AIM1L	0.0384	1.15
Transglutaminase 4	TGM4	0.0312	1.15

DSN1 homolog, MIS12 kinetochore complex component	DSN1	0.0238	1.15
KIAA0355	KIAA0355	0.0191	1.15
Coiled-coil domain containing 162, pseudogene	CCDC162P	0.0343	1.15
Armadillo repeat containing, X-linked 2	ARMCX2	0.0217	1.15
Arrestin domain containing 2	ARRDC2	0.0316	1.14
Solute carrier family 22, member 24	SLC22A24	0.0415	1.14
Intelectin 2	ITLN2	0.0097	1.14
Ras association (ralgds	RASSF3	0.0259	1.14
Transcript Identified by aceview, Entrez Gene ID(s) 5	PTK2	0.0271	1.14
Centrosome and spindle pole associated protein 1	CSPP1	0.0342	1.14
Heparin-binding EGF-like growth factor	HBEGF	0.0420	1.14
Methyl-cpg binding domain protein 1	MBD1	0.0297	1.14
Elongation factor RNA polymerase II-like 3	ELL3	0.0479	1.14
Methyltransferase like 16	METTL16	0.0246	1.14
WD repeat domain 83 opposite strand	WDR83OS	0.0463	1.14
Sad1 and UNC84 domain containing 2	SUN2	0.0305	1.14
Death effector domain containing 2	DEDD2	0.0254	1.14
TSEN15 trna splicing endonuclease subunit	TSEN15	0.0291	1.14
Calcium binding protein 4	CABP4	0.0212	1.14
Autoimmune regulator	AIRE	0.0220	1.14
Transcription factor 20 (AR1)	TCF20	0.0313	1.14
Transcription factor 20 (AR1)	TCF20	0.0313	1.14
3-hydroxyacyl-coa dehydratase 1	HACD1	0.0300	1.14
Leucine rich repeat containing 23	LRRC23	0.0127	1.14
Complement component 3	C3	0.0402	1.14
Calcium channel, voltage-dependent, alpha 2	CACNA2D2	0.0307	1.14
Chondroitin polymerizing factor 2	CHPF2	0.0072	1.14
Terminal uridylyl transferase 1, U6 snrna-specific	TUT1	0.0309	1.14
Acyl-coa synthetase medium-chain family member 2B	ACSM2B	0.0329	1.14
Surfeit 6	SURF6	0.0360	1.14
Chymotrypsin-like elastase family, member 3B	CELA3B	0.0415	1.14
Diacylglycerol kinase, delta 130kda	DGKD	0.0362	1.14

Mitochondrial ribosomal protein S16	MRPS16	0.0230	1.14
Sp6 transcription factor	SP6	0.0120	1.14
Ring finger protein 186	RNF186	0.0257	1.14
Uncharacterized LOC102724957	LOC102724957	0.0445	1.14
Claudin 34	CLDN34	0.0146	1.14
Transmembrane protein 173	TMEM173	0.0305	1.14
Chromosome 11 open reading frame 68	C11orf68	0.0289	1.14
Polymerase (DNA directed), mu	POLM	0.0318	1.14
Platelet-activating factor acetylhydrolase 1b, catalytic su	PAFAH1B3	0.0289	1.14
Zinc finger protein 460	ZNF460	0.0180	1.14
Ubiquitin specific peptidase 17-like family member 2	USP17L2	0.0299	1.14
Tripartite motif family like 1	TRIML1	0.0163	1.14
Ribosomal protein S25	RPS25	0.0190	1.14
Angel homolog 2 (Drosophila)	ANGEL2	0.0467	1.14
Solute carrier family 35 (UDP-glca	SLC35D1	0.0443	1.14
Chromosome 8 open reading frame 76	C8orf76	0.0343	1.14
BAI1-associated protein 2	BAIAP2	0.0117	1.14
N-terminal EF-hand calcium binding protein 3	NECAB3	0.0059	1.14
Proteasome inhibitor subunit 1	PSMF1	0.0298	1.14
Nudix hydrolase 4	NUDT4	0.0313	1.14
DND microrna-mediated repression inhibitor 1	DND1	0.0274	1.14
Sarcolipin	SLN	0.0401	1.14
Leukocyte immunoglobulin-like receptor, subfamily B (with	LILRB1	0.0499	1.14
DDRGK domain containing 1	DDRGK1	0.0347	1.14
Chromosome 1 open reading frame 87	C1orf87	0.0382	1.14
ADAM metallopeptidase with thrombospondin type 1 motif 4	ADAMTS4	0.0482	1.14
Forkhead box E3	FOXE3	0.0172	1.14
PRAME family member 10	PRAMEF10	0.0427	1.14
Spermidine	SATL1	0.0407	1.13
Small muscle protein, X-linked	SMPX	0.0418	1.13
Heterogeneous nuclear ribonucleoprotein A	HNRNPAB	0.0485	1.13
1-acylglycerol-3-phosphate O-acyltransferase 3	AGPAT3	0.0441	1.13
Phosducin like 3	PDCL3	0.0296	1.13
RNA binding motif (RNP1, RRM) protein 3	RBM3	0.0326	1.13

Carboxypeptidase N, polypeptide 1	CPN1	0.0418	1.13
Guanylate binding protein 7	GBP7	0.0444	1.13
Free fatty acid receptor 2	FFAR2	0.0168	1.13
Pseudouridylate synthase 1	PUS1	0.0498	1.13
Apelin receptor	APLNR	0.0310	1.13
Transmembrane protein 14B	TMEM14B	0.0391	1.13
TSPY-like 5	TSPYL5	0.0305	1.13
Uncharacterized LOC728485	LOC728485	0.0367	1.13
Synuclein gamma	SNCG	0.0477	1.13
3-oxoacid coa-transferase 2 pseudogene 1	OXCT2P1	0.0315	1.13
Neuronatin	NNAT	0.0447	1.13
Chromosome X open reading frame 38	CXorf38	0.0462	1.13
MT-RNR2-like 7	MTRNR2L7	0.0362	1.13
Plexin B2	PLXNB2	0.0465	1.13
Vomeronasal 1 receptor 1	VN1R1	0.0141	1.13
C-type lectin domain family 1, member B	CLEC1B	0.0264	1.13
Cyclin Pas1	CNPPD1	0.0431	1.13
Atonal bhlh transcription factor 1	ATOH1	0.0347	1.13
DEXH (Asp-Glu-X-His) box polypeptide 58	DHX58	0.0224	1.13
Solute carrier family 19 (folate transporter), member 1	SLC19A1	0.0151	1.13
MAGE family member A12	MAGEA12	0.0411	1.13
Forkhead box L1-like	WI2-2373I1.2	0.0363	1.13
Visinin like 1	VSNL1	0.0446	1.13
Olfactory receptor, family 2, subfamily J, member 1 (gen	OR2J1	0.0226	1.13
Thrombospondin type 1 domain containing 7B	THSD7B	0.0493	1.13
Reversion-inducing-cysteine-rich protein with kazal motifs	RECK	0.0219	1.13
Adaptor-related protein complex 3, mu 2 subunit	AP3M2	0.0338	1.13
Family with sequence similarity 200, member B	FAM200B	0.0421	1.13
Pleckstrin homology-like domain, family A, member 3	PHLDA3	0.0196	1.13
Anaphase promoting complex subunit 11	ANAPC11	0.0181	1.13
Transmembrane and coiled-coil domains 1	TMCO1	0.0270	1.13
Ribosomal protein S6 kinase, 70kda, polypeptide 2	RPS6KB2	0.0355	1.13
Perilipin 3	PLIN3	0.0268	1.12

PRKC, apoptosis, WT1, regulator	PAWR	0.0246	1.12
Zinc finger protein 227	ZNF227	0.0476	1.12
Tyrosine 3-monooxygenase	YWHAQ	0.0166	1.12
FERM domain containing 6	FRMD6	0.0343	1.12
Gremlin 1, DAN family BMP antagonist	GREM1	0.0454	1.12
O-sialoglycoprotein endopeptidase	OSGEP	0.0492	1.12
Olfactory receptor, family 52, subfamily I, member 1	OR52I1	0.0379	1.12
Ubiquinol-cytochrome c reductase hinge protein	UQCRH	0.0416	1.12
TBC1 domain family, member 28	TBC1D28	0.0436	1.12
Neural retina leucine zipper	NRL	0.0400	1.12
Lectin, mannose-binding 2-like	LMAN2L	0.0447	1.12
Phospholipid scramblase 2	PLSCR2	0.0240	1.12
Zhang2013 ALT_ACCEPTOR, ALT_DONOR, coding, INTERNAL, intron	SNX5	0.0455	1.12
Synthetic construct Homo sapiens clone IMAGE:100069019, MGC:19903	MBD3L2	0.0431	1.12
Myotubularin related protein 8	MTMR8	0.0344	1.12
Centrosomal protein 97kda	CEP97	0.0434	1.12
Bobby sox homolog (Drosophila)	BBX	0.0492	1.12
Arachidonate 15-lipoxygenase	ALOX15	0.0326	1.12
Zinc finger protein 391	ZNF391	0.0360	1.12
Aprataxin and PNKP like factor	APLF	0.0250	1.12
Kyphoscoliosis peptidase	KY	0.0450	1.12
PCF11 cleavage and polyadenylation factor subunit	PCF11	0.0369	1.12
UDP glucuronosyltransferase 2 family, polypeptide B7	UGT2B7	0.0372	1.12
G protein pathway suppressor 1	GPS1	0.0282	1.12
Ret finger protein-like 3	RFPL3	0.0305	1.12
CDC42 effector protein (Rho gtpase binding) 1	CDC42EP1	0.0389	1.12
Tripartite motif family like 2	TRIML2	0.0255	1.12
Apolipoprotein C-II	APOC2	0.0329	1.12
UBX domain protein 1	UBXN1	0.0167	1.12
FIC domain containing	FICD	0.0243	1.12
GATA zinc finger domain containing 1	GATAD1	0.0154	1.12
Family with sequence similarity 179, member B	FAM179B	0.0397	1.12
Emopamil binding protein (sterol isomerase)	EBP	0.0490	1.11
Ring finger protein 148	RNF148	0.0370	1.11

U2 small nuclear RNA auxiliary factor 1	U2AF1	0.0404	1.11
Neuronal tyrosine-phosphorylated phosphoinositide-3-kinase adapto	NYAP1	0.0180	1.11
Testis specific 10	TSGA10	0.0241	1.11
Ubiquitin domain containing 1	UBTD1	0.0366	1.11
Ubiquitin conjugating enzyme E2A	UBE2A	0.0228	1.11
DEAD (Asp-Glu-Ala-Asp) box helicase 42	DDX42	0.0472	1.11
Abl-interactor 2	ABI2	0.0052	1.11
Keratin 1, type II	KRT1	0.0430	1.11
Glutamate rich 6B	ERICH6B	0.0292	1.11
Slit guidance ligand 2	SLIT2	0.0271	1.11
Ets variant 4	ETV4	0.0392	1.11
Gamma-glutamyl carboxylase	GGCX	0.0333	1.11
Tripartite motif containing 17	TRIM17	0.0373	1.11
Dual specificity tyrosine-(Y)-phosphorylation regulated kinase	DYRK3	0.0420	1.10
KIAA1109	KIAA1109	0.0493	1.10
Coatomer protein complex subunit beta 1	COPB1	0.0175	1.10
TATA box binding protein like 2	TBPL2	0.0412	1.10
WW domain containing E3 ubiquitin protein ligase 1	WWP1	0.0099	1.10
Achaete-scute family bhlh transcription factor 4	ASCL4	0.0195	1.10
VMA21 vacuolar H+-atpase homolog (S. Cerevisiae)	VMA21	0.0208	1.10
Primase, DNA, polypeptide 2 (58kda)	PRIM2	0.0404	1.10
Family with sequence similarity 234, member B	FAM234B	0.0212	1.10
Epoxide hydrolase 2, cytoplasmic	EPHX2	0.0353	1.10
NPHP3-ACAD11 readthrough (NMD candidate)	NPHP3-ACAD11	0.0382	1.10
Katanin p60 subunit A-like 1	KATNAL1	0.0451	1.10
MAGE family member A9B	MAGEA9B	0.0499	1.10
Oligodendrocyte myelin glycoprotein	OMG	0.0338	1.10
Cell division cycle 42	CDC42	0.0315	1.09
Olfactory receptor, family 10, subfamily V, member 1	OR10V1	0.0260	1.09
Keratin associated protein 13-4	KRTAP13-4	0.0245	1.09
HSPE1-MOB4 readthrough	HSPE1-MOB4	0.0374	1.08
CD58 molecule	CD58	0.0478	-1.07
Family with sequence similarity 63, member B	FAM63B	0.0447	-1.07

Olfactory receptor, family 56, subfamily A, member 3	OR56A3	0.0237	-1.07
HORMA domain containing 2	HORMAD2	0.0355	-1.07
Hornerin	HRNR	0.0448	-1.07
Alkaline ceramidase 3	ACER3	0.0417	-1.07
Rho gtpase activating protein 11B	ARHGAP11B	0.0432	-1.08
NSFL1 (p97) cofactor (p47)	NSFL1C	0.0277	-1.08
General transcription factor IIIA	GTF3A	0.0378	-1.08
Family with sequence similarity 177, member A1	FAM177A1	0.0457	-1.08
MCF.2 cell line derived transforming sequence-like 2	MCF2L2	0.0490	-1.08
Extended synaptotagmin-like protein 2	ESYT2	0.0415	-1.08
Kelch-like family member 9	KLHL9	0.0477	-1.08
Coiled-coil domain containing 88C	CCDC88C	0.0417	-1.09
Amphiregulin	AREG	0.0349	-1.09
NLR family, apoptosis inhibitory protein	NAIP	0.0288	-1.09
KIAA0101	KIAA0101	0.0390	-1.09
Cleavage and polyadenylation specific factor 4-like	CPSF4L	0.0458	-1.09
Olfactory receptor, family 51, subfamily F, member 2	OR51F2	0.0347	-1.09
EF-hand calcium binding domain 13	EFCAB13	0.0343	-1.09
Protein tyrosine phosphatase, non-receptor type 11	PTPN11	0.0424	-1.09
Transmembrane protein 50A	TMEM50A	0.0389	-1.09
Zinc finger protein 546	ZNF546	0.0327	-1.09
ALG11, alpha-1,2-mannosyltransferase	ALG11	0.0269	-1.09
Ribosomal protein L9 pseudogene 7	RPL9P7	0.0296	-1.09
Chromosome 5 open reading frame 30	C5orf30	0.0325	-1.09
Olfactory receptor, family 11, subfamily H, member 6	OR11H6	0.0387	-1.09
Tudor domain containing 5	TDRD5	0.0296	-1.09
Tubulin, delta 1	TUBD1	0.0147	-1.09
Transcription factor A, mitochondrial	TFAM	0.0362	-1.09
Integrator complex subunit 8	INTS8	0.0499	-1.09
Zinc finger and BTB domain containing 32	ZBTB32	0.0493	-1.09
T cell receptor associated transmembrane adaptor 1	TRAT1	0.0316	-1.09
Transcript Identified by aceview, Entrez Gene	RAD51AP2	0.0455	-1.09

Aquaporin 11	AQP11	0.0077	-1.09
Ras homolog enriched in brain	RHEB	0.0143	-1.10
Transmembrane protein 30A	TMEM30A	0.0376	-1.10
Signal transducer and activator of transcription 4	STAT4	0.0265	-1.10
Zinc activated ligand-gated ion channel	ZACN	0.0397	-1.10
Ubiquitin-fold modifier 1	UFM1	0.0442	-1.10
Thyroid hormone receptor interactor 11	TRIP11	0.0490	-1.10
POTE ankyrin domain family, member D	POTED	0.0156	-1.10
Utrophin	UTRN	0.0381	-1.10
Centrosomal protein 83kda	CEP83	0.0070	-1.10
Proteasome 26S subunit, non-atpase 1	PSMD1	0.0312	-1.10
Glucosaminyl (N-acetyl) transferase 2, I-branching enzyme (I bloo	GCNT2	0.0483	-1.10
Nucleotide-binding oligomerization domain containing 2	NOD2	0.0495	-1.10
Defensin, beta 121	DEFB121	0.0416	-1.10
Naked cuticle homolog 1 (Drosophila)	NKD1	0.0487	-1.10
Tubby like protein 4	TULP4	0.0469	-1.10
Membrane associated ring finger 8	MARCH8	0.0116	-1.10
F-box protein 6	FBXO6	0.0246	-1.10
Macrophage scavenger receptor 1	MSR1	0.0448	-1.10
Chromosome 10 open reading frame 107	C10orf107	0.0124	-1.10
Ermin	ERMN	0.0316	-1.10
WD repeat domain 66	WDR66	0.0485	-1.10
G protein-coupled receptor 89A	GPR89A	0.0362	-1.10
Deleted in azoospermia 4	DAZ4	0.0432	-1.10
LMBR1 domain containing 2	LMBRD2	0.0267	-1.10
Coiled-coil domain containing 89	CCDC89	0.0484	-1.10
Coiled-coil domain containing 84	CCDC84	0.0485	-1.10
NMD3 ribosome export adaptor	NMD3	0.0150	-1.10
HLA complex group 22	HCG22	0.0151	-1.11
Ankyrin repeat domain 62	ANKRD62	0.0455	-1.11
Centrosomal protein 290kda	CEP290	0.0284	-1.11
Kinesin family member 3A	KIF3A	0.0355	-1.11
Urotensin 2	UTS2	0.0343	-1.11
Astrotactin 2	ASTN2	0.0171	-1.11
Eukaryotic translation initiation factor 1B	EIF1B	0.0485	-1.11
Myosin, heavy chain 10, non-muscle	MYH10	0.0284	-1.11
Numb homolog (Drosophila)-like	NUMBL	0.0498	-1.11

Mediator complex subunit 21	MED21	0.0314	-1.11
Nuclear factor of kappa light polypeptide gene enhancer in B-	NFKBIZ	0.0447	-1.11
SMC5-SMC6 complex localization factor 1	SLF1	0.0448	-1.11
EF-hand calcium binding domain 5	EFCAB5	0.0154	-1.11
Transmembrane phosphatase with tensin homology	TPTE	0.0122	-1.11
Zhang2013 ALT_DONOR, coding, INTERNAL, intronic, OVERLAPTX	LARP7	0.0459	-1.11
Topoisomerase (DNA) II binding protein 1	TOPBP1	0.0488	-1.11
Zinc finger protein 502	ZNF502	0.0425	-1.11
Autophagy	AMBRA1	0.0345	-1.11
LSM3 homolog, U6 small nuclear RNA and mrna degradation associated	LSM3	0.0472	-1.11
Kinetochore associated 1	KNTC1	0.0240	-1.11
Methionyl aminopeptidase 1	METAP1	0.0049	-1.11
Solute carrier family 38, member 4	SLC38A4	0.0175	-1.11
Salt-inducible kinase 1	SIK1	0.0195	-1.11
Integrin beta 3 binding protein (beta3-endonexin)	ITGB3BP	0.0205	-1.11
Prostaglandin E synthase 3 (cytosolic)	PTGES3	0.0405	-1.11
NADH dehydrogenase (ubiquinone) complex I, assembly factor 6	NDUFAF6	0.0151	-1.11
Pleckstrin homology domain containing, family G (with rhogef	PLEKHG7	0.0432	-1.11
Star-related lipid transfer domain containing 4	STARD4	0.0442	-1.11
Chromobox homolog 7	CBX7	0.0326	-1.11
Nucleolar protein 8	NOL8	0.0144	-1.11
Ring finger protein 133	RNF133	0.0269	-1.11
Growth hormone receptor	GHR	0.0323	-1.11
Ankyrin repeat domain 18B	ANKRD18B	0.0058	-1.11
Acyl-coa thioesterase 12	ACOT12	0.0192	-1.11
Glycophorin B (MNS blood group)	GYPB	0.0102	-1.11
EF-hand calcium binding domain 2	EFCAB2	0.0402	-1.11
Interferon-induced protein 44	IFI44	0.0227	-1.11
CWF19-like 2, cell cycle control (S. Pombe)	CWF19L2	0.0258	-1.11
NUF2, NDC80 kinetochore complex component	NUF2	0.0462	-1.11
Ecotropic viral integration site 5	EVI5	0.0373	-1.11
HECT domain and ankyrin repeat containing E3 ubiquitin protein li	HACE1	0.0422	-1.11

Taste receptor, type 2, member 38	TAS2R38	0.0271	-1.11
Zinc finger protein 365	ZNF365	0.0459	-1.11
Coiled-coil domain containing 50	CCDC50	0.0458	-1.11
Protein tyrosine phosphatase, non-receptor type 2	PTPN2	0.0171	-1.11
Cyclin-dependent kinase 19	CDK19	0.0184	-1.11
HECT domain containing E3 ubiquitin protein ligase 2	HECTD2	0.0134	-1.11
SPATA31 subfamily D, member 4	SPATA31D4	0.0480	-1.11
Trichoplein, keratin filament binding	TCHP	0.0364	-1.11
Wolfram syndrome 1 (wolframin)	WFS1	0.0496	-1.11
Enoyl-coa, hydratase	EHHADH	0.0490	-1.11
Chromosome 6 open reading frame 222	C6orf222	0.0416	-1.11
Nicotinamide riboside kinase 1	NMRK1	0.0037	-1.11
Ubiquitin specific peptidase 17-like family member 28	USP17L28	0.0154	-1.11
Lysosomal-associated membrane protein 2	LAMP2	0.0215	-1.11
ADAM metallopeptidase domain 21	ADAM21	0.0440	-1.11
Memczak2013 ANTISENSE, coding, INTERNAL, intronic best tr	ADAM28	0.0387	-1.11
Olfactory receptor, family 5, subfamily P, member 2	OR5P2	0.0082	-1.11
Uncharacterized LOC100130691	LOC100130691	0.0394	-1.11
Activating transcription factor 7 interacting protein 2	ATF7IP2	0.0314	-1.11
Carbonic anhydrase VIII	CA8	0.0148	-1.11
MIS12 kinetochore complex component	MIS12	0.0476	-1.11
SNF2 histone linker PHD RING helicase, E3 ubiquitin protein li	SHPRH	0.0314	-1.11
G protein-coupled receptor 65	GPR65	0.0317	-1.11
Syntrophin, beta 1 (dystrophin-associated protein A1, 59kda, basi	SNTB1	0.0479	-1.11
Chromosome 15 open reading frame 40	C15orf40	0.0467	-1.11
Relaxin	RXFP2	0.0051	-1.12
Zinc finger protein 383	ZNF383	0.0280	-1.12
Chromosome 12 open reading frame 66	C12orf66	0.0196	-1.12
NOP2	NSUN6	0.0457	-1.12
Transducin (beta)-like 1 X-linked receptor 1	TBL1XR1	0.0121	-1.12
N-deacetylase	NDST3	0.0363	-1.12
N(alpha)-acetyltransferase 35, natc auxiliary subunit	NAA35	0.0011	-1.12

Neuron navigator 3	NAV3	0.0298	-1.12
Olfactory receptor, family 8, subfamily B, member 3	OR8B3	0.0088	-1.12
Uncharacterized LOC151121	LOC151121	0.0242	-1.12
Jeck2013 ALT_ACCEPTOR, ALT_DONOR, coding, INTERNAL, intron	AGAP1	0.0345	-1.12
Guanylate binding protein 2, interferon-inducible	GBP2	0.0336	-1.12
Heat shock transcription factor 2	HSF2	0.0233	-1.12
Solute carrier family 16 (monocarboxylate transporter), m	SLC16A1	0.0099	-1.12
T brachyury transcription factor	T	0.0226	-1.12
RAD18 E3 ubiquitin protein ligase	RAD18	0.0425	-1.12
ADAM metallopeptidase domain 28	ADAM28	0.0178	-1.12
Zinc finger, HIT-type containing 6	ZNHIT6	0.0095	-1.12
TBC1 domain family, member 23	TBC1D23	0.0316	-1.12
Arginyltransferase 1	ATE1	0.0313	-1.12
Endothelial differentiation-related factor 1	EDF1	0.0474	-1.12
Secernin 1	SCRN1	0.0218	-1.12
Retinoblastoma binding protein 5	RBBP5	0.0346	-1.12
Transmembrane protein 255A	TMEM255A	0.0357	-1.12
CTAGE family, member 5	CTAGE5	0.0297	-1.12
CCDC144NL antisense RNA 1	CCDC144NL-AS1	0.0250	-1.12
Diacylglycerol kinase, epsilon 64kda	DGKE	0.0273	-1.12
Zinc finger protein 223	ZNF223	0.0421	-1.12
Mucolipin 2	MCOLN2	0.0296	-1.12
Ligase IV, DNA, ATP-dependent	LIG4	0.0371	-1.12
Zinc finger protein 224	ZNF224	0.0380	-1.12
Arfgap with gtpase domain, ankyrin repeat and PH domain 9	AGAP9	0.0302	-1.12
Chromosome 5 open reading frame 58	C5orf58	0.0313	-1.12
Zinc finger protein 181	ZNF181	0.0214	-1.12
SET domain, bifurcated 2	SETDB2	0.0154	-1.12
Regulator of cell cycle	RGCC	0.0186	-1.12
Cysteine rich with EGF-like domains 2	CRELD2	0.0203	-1.12
Neurobeachin like 1	NBEAL1	0.0112	-1.12
Vacuolar protein sorting 51 homolog (S. Cerevisiae)	VPS51	0.0346	-1.12
Alpha thalassemia	ATRX	0.0088	-1.12
Ubiquitin-like domain containing CTD phosphatase 1	UBLCP1	0.0362	-1.12
Leucine rich repeat containing 40	LRRC40	0.0464	-1.12
Claudin 2	CLDN2	0.0393	-1.12

Golgin A8 family, member A	GOLGA8A	0.0441	-1.12
DCN1, defective in cullin neddylation 1, domain containing 3	DCUN1D3	0.0410	-1.12
Zinc finger protein 780A	ZNF780A	0.0193	-1.12
RAN binding protein 6	RANBP6	0.0310	-1.12
Brain expressed X-linked 2	BEX2	0.0410	-1.12
Transmembrane protein 208	TMEM208	0.0449	-1.12
CD99 molecule-like 2	CD99L2	0.0308	-1.12
Protein phosphatase 2, regulatory subunit B, alpha	PPP2R2A	0.0449	-1.12
Family with sequence similarity 160, member B2	FAM160B2	0.0394	-1.12
Cytochrome c oxidase subunit VIIIC	COX8C	0.0427	-1.12
Thiosulfate sulfurtransferase (rhodanese)-like domain containi	TSTD3	0.0259	-1.12
SLAM family member 7	SLAMF7	0.0233	-1.12
Transcript Identified by aceview	boyboy	0.0287	-1.12
Zinc finger protein 136	ZNF136	0.0174	-1.12
TBC1 domain family, member 31	TBC1D31	0.0219	-1.13
Breast cancer metastasis-suppressor 1-like	BRMS1L	0.0260	-1.13
MAS-related GPR, member X4	MRGPRX4	0.0453	-1.13
LLP homolog, long-term synaptic facilitation (Aplysia)	LLPH	0.0299	-1.13
INO80B-WBP1 readthrough (NMD candidate)	INO80B-WBP1	0.0155	-1.13
Castor zinc finger 1	CASZ1	0.0444	-1.13
BCL2-like 14 (apoptosis facilitator)	BCL2L14	0.0473	-1.13
Retinoblastoma binding protein 7	RBBP7	0.0467	-1.13
Transcription elongation factor A (SII)-like 6	TCEAL6	0.0380	-1.13
Coiled-coil domain containing 102B	CCDC102B	0.0056	-1.13
Zinc finger protein 532	ZNF532	0.0378	-1.13
Transcript Identified by aceview, Entrez Gene ID(s) 52	PHKB	0.0279	-1.13
Trafficking protein particle complex 4	TRAPPC4	0.0278	-1.13
DIS3 like 3-5 exoribonuclease 2	DIS3L2	0.0029	-1.13
GIPC PDZ domain containing family, member 1	GIPC1	0.0479	-1.13
Bbsome interacting protein 1	BBIP1	0.0371	-1.13
ST20-MTHFS readthrough	ST20-MTHFS	0.0253	-1.13
TBC1 domain family, member 8 (with GRAM domain)	TBC1D8	0.0326	-1.13
Olfactory receptor, family 4, subfamily C, member 45	OR4C45	0.0159	-1.13
Dual specificity phosphatase 19	DUSP19	0.0150	-1.13

Zinc finger protein 483	ZNF483	0.0181	-1.13
Polymerase (DNA directed), theta	POLQ	0.0469	-1.13
Family with sequence similarity 46, member A	FAM46A	0.0107	-1.13
Olfactory receptor, family 2, subfamily M, member 7	OR2M7	0.0303	-1.13
Complement component 1, q subcomponent-like 3	C1QL3	0.0202	-1.13
Aprataxin	APTX	0.0141	-1.13
Nuclear RNA export factor 2B	NXF2B	0.0074	-1.13
Serine peptidase inhibitor, Kazal type 8 (putative)	SPINK8	0.0017	-1.13
Transcription elongation factor A (SII)-like 3	TCEAL3	0.0454	-1.13
Keratin 24, type I	KRT24	0.0385	-1.13
Cystic fibrosis transmembrane conductance regulator	CFTR	0.0047	-1.13
ELL associated factor 2	EAF2	0.0356	-1.13
Cilia and flagella associated protein 61	CFAP61	0.0178	-1.13
La ribonucleoprotein domain family, member 1	LARP1	0.0467	-1.13
Long intergenic non-protein coding RNA 174	LINC00174	0.0081	-1.13
Mannose receptor, C type 2	MRC2	0.0193	-1.13
Transmembrane protein 78	TMEM78	0.0220	-1.13
Protease, serine 35	PRSS35	0.0370	-1.13
Zinc finger protein 239	ZNF239	0.0142	-1.13
Eukaryotic translation initiation factor 2-alpha kinase 2	EIF2AK2	0.0146	-1.13
Transcript Identified by aceview, Entrez Gene ID(s) 8453	CUL2	0.0314	-1.13
Family with sequence similarity 186, member A	FAM186A	0.0301	-1.13
Transcript Identified by aceview, Entrez Gene ID(s) 78	EVI5	0.0317	-1.13
Aminoacyl trna synthetase complex-interacting multifunctional	AIMP1	0.0286	-1.13
RANBP2-like and GRIP domain containing 2	RGPD2	0.0136	-1.13
Proprotein convertase subtilisin	PCSK5	0.0486	-1.13
Kv channel interacting protein 3, calsenilin	KCNIP3	0.0184	-1.13
Interleukin 7	IL7	0.0134	-1.13
Intraflagellar transport 46	IFT46	0.0081	-1.13
Tryptophan hydroxylase 2	TPH2	0.0253	-1.13
F-box protein 38	FBXO38	0.0192	-1.13

Coiled-coil domain containing 66	CCDC66	0.0060	-1.13
Metastasis associated in colon cancer 1	MACC1	0.0407	-1.13
TP53 induced glycolysis regulatory phosphatase	TIGAR	0.0282	-1.13
Huntingtin interacting protein K	HYPK	0.0463	-1.13
Polycystic kidney and hepatic disease 1 (autosomal recessive)-l	PKHD1L1	0.0048	-1.13
Ring finger protein 25	RNF25	0.0405	-1.13
TBCC domain containing 1	TBCCD1	0.0380	-1.13
G2	G2E3	0.0032	-1.13
Myeloid	MLLT4	0.0435	-1.13
Melanoma inhibitory activity	MIA	0.0342	-1.13
Xin actin binding repeat containing 2	XIRP2	0.0167	-1.13
Heat shock 10kda protein 1	HSPE1	0.0438	-1.13
Olfactory receptor, family 1, subfamily L, member 8	OR1L8	0.0442	-1.13
3-hydroxyisobutyryl-coa hydrolase	HIBCH	0.0255	-1.13
THO complex 2	THOC2	0.0491	-1.13
Anaphase promoting complex subunit 13	ANAPC13	0.0285	-1.13
Integrin linked kinase	ILK	0.0388	-1.13
Ras association (ralgds	RASSF9	0.0229	-1.13
Integrator complex subunit 12	INTS12	0.0196	-1.13
UDP-glucose glycoprotein glucosyltransferase 2	UGGT2	0.0239	-1.13
Spermatogenesis associated, serine-rich 2	SPATS2	0.0302	-1.13
FYVE, rhogef and PH domain containing 3	FGD3	0.0452	-1.13
Salt-inducible kinase 2	SIK2	0.0219	-1.13
Guanine nucleotide binding protein (G protein), beta polypeptid	GNB3	0.0324	-1.13
Memczak2013 ALT_ACCEPTOR, ALT_DONOR, coding, INTERNAL, int	XRCC5	0.0034	-1.13
Ras and Rab interactor like	RINL	0.0251	-1.13
ADP-ribosylation factor like gtpase 9	ARL9	0.0378	-1.14
SUMO1	SENP7	0.0158	-1.14
Vacuolar protein sorting 45 homolog (S. Cerevisiae)	VPS45	0.0295	-1.14
Zinc finger and BTB domain containing 7A	ZBTB7A	0.0488	-1.14
Transcript Identified by aceview, Entrez Gene ID(	ARPP21	0.0497	-1.14
Myeloperoxidase	MPO	0.0446	-1.14
Disrupted in renal carcinoma 2	DIRC2	0.0232	-1.14

Solute carrier family 35 (UDP-N-acetylglucosamine (UDP-glcna	SLC35A3	0.0348	-1.14
Family with sequence similarity 177, member A1	FAM177A1	0.0098	-1.14
Protein phosphatase 1, regulatory subunit 37	PPP1R37	0.0388	-1.14
Multiple C2 domains, transmembrane 2	MCTP2	0.0181	-1.14
Zinc finger, AN1-type domain 1	ZFAND1	0.0469	-1.14
Coiled-coil domain containing 96	CCDC96	0.0222	-1.14
Interferon, alpha 4	IFNA4	0.0294	-1.14
Transcript Identified by aceview, Entrez Gene ID(s) 79	SMC6	0.0260	-1.14
Trace amine associated receptor 8	TAAR8	0.0487	-1.14
Dnaj (Hsp40) homolog, subfamily C, member 12	DNAJC12	0.0366	-1.14
Long intergenic non-protein coding RNA 1496	LINC01496	0.0297	-1.14
Chromodomain helicase DNA binding protein 9	CHD9	0.0351	-1.14
Mediator complex subunit 7	MED7	0.0481	-1.14
Potassium channel, voltage gated eag related subfamily H, member	KCNH7	0.0426	-1.14
TSPY-like 2	TSPYL2	0.0251	-1.14
MANSC domain containing 4	MANSC4	0.0242	-1.14
Karyopherin alpha 3 (importin alpha 4)	KPNA3	0.0346	-1.14
Ring finger protein 168, E3 ubiquitin protein ligase	RNF168	0.0261	-1.14
Olfactory receptor, family 10, subfamily A, member 3	OR10A3	0.0130	-1.14
Nuclear factor of kappa light polypeptide gene enhancer in B-c	NFKB1	0.0354	-1.14
Protein prenyltransferase alpha subunit repeat containing 1	PTAR1	0.0246	-1.14
Ribosomal RNA adenine dimethylase domain containing 1	RRNAD1	0.0178	-1.14
Cytochrome P450, family 3, subfamily A, polypeptide 7	CYP3A7	0.0147	-1.14
Zinc finger protein 275	ZNF275	0.0312	-1.14
Spectrin, beta, non-erythrocytic 2	SPTBN2	0.0377	-1.14
Zhang2013 ALT_ACCEPTOR, ALT_DONOR, coding, INTERNAL, intro	EXOC7	0.0385	-1.14
POC5 centriolar protein	POC5	0.0050	-1.14
Chromosome 20 open reading frame 197	C20orf197	0.0427	-1.14

GPN-loop gtpase 1	GPN1	0.0371	-1.14
Interferon regulatory factor 6	IRF6	0.0233	-1.14
Trna methyltransferase 6	TRMT6	0.0197	-1.14
SWT1 RNA endoribonuclease homolog	SWT1	0.0181	-1.14
Transmembrane protein 126A	TMEM126A	0.0320	-1.14
LIM homeobox 1	LHX1	0.0372	-1.14
Olfactory receptor, family 2, subfamily B, member 3	OR2B3	0.0049	-1.14
Chromosome 12 open reading frame 42	C12orf42	0.0233	-1.14
Endogenous retrovirus group V, member 1	ERVV-1	0.0047	-1.14
Cold shock domain containing C2, RNA binding	CSDC2	0.0222	-1.14
Olfactory receptor, family 2, subfamily G, member 6	OR2G6	0.0308	-1.14
Calcitonin-related polypeptide beta	CALCB	0.0385	-1.14
POU class 1 homeobox 1	POU1F1	0.0343	-1.14
Thymosin beta 15a	TMSB15A	0.0222	-1.14
Killer cell lectin-like receptor subfamily A pseudogene 1	KLRAP1	0.0197	-1.14
Chromosome 9 open reading frame 92	C9orf92	0.0054	-1.14
SP100 nuclear antigen	SP100	0.0221	-1.14
Tetratricopeptide repeat domain 21B	TTC21B	0.0048	-1.14
CCR4-NOT transcription complex subunit 6	CNOT6	0.0205	-1.14
EGF containing fibulin-like extracellular matrix protein 1	EFEMP1	0.0287	-1.14
Adaptor-related protein complex 4, epsilon 1 subunit	AP4E1	0.0113	-1.14
Dihydropyrimidinase	DPYS	0.0337	-1.14
Transcript Identified by aceview, Entrez Gene ID(s)	TM2D1	0.0386	-1.14
Protein phosphatase, Mg2+	PPM1B	0.0169	-1.14
SH3-domain GRB2-like endophilin B1	SH3GLB1	0.0490	-1.14
KH homology domain containing 1	KHDC1	0.0313	-1.14
Memczak2013 ANTISENSE, coding, INTERNAL, intronic best tr	DLGAP1	0.0121	-1.14
Ribonuclease, rnase A family, 1 (pancreatic)	RNASE1	0.0492	-1.14
Membrane protein, palmitoylated 6	MPP6	0.0291	-1.14
ORMDL sphingolipid biosynthesis regulator 3	ORMDL3	0.0090	-1.14

Matrix metallopeptidase 27	MMP27	0.0095	-1.14
Kinesin family member 15	KIF15	0.0216	-1.14
LDL receptor related protein 6	LRP6	0.0241	-1.14
Trna methyltransferase 12 homolog (S. Cerevisiae)	TRMT12	0.0326	-1.14
Cyclin-dependent kinase inhibitor 1B (p27, Kip1)	CDKN1B	0.0321	-1.14
Sorting nexin 24	SNX24	0.0350	-1.14
Glutamate receptor, ionotropic, delta 1	GRID1	0.0463	-1.14
Additional sex combs like transcriptional regulator 2	ASXL2	0.0476	-1.14
Family with sequence similarity 19 (chemokine (C-C motif)-li	FAM19A3	0.0204	-1.14
Keratin associated protein 20-4	KRTAP20-4	0.0473	-1.14
Mannosidase, endo-alpha	MANEA	0.0379	-1.14
Polymerase (DNA directed) kappa	POLK	0.0496	-1.14
Zinc finger, CW type with PWWP domain 1	ZCWPW1	0.0162	-1.14
Transmembrane 4 L six family member 18	TM4SF18	0.0179	-1.14
Retinal pigment epithelium-specific protein 65kda	RPE65	0.0318	-1.14
SEC24 homolog B, COPII coat complex component	SEC24B	0.0442	-1.14
Regulator of G-protein signaling 4	RGS4	0.0493	-1.14
Cytochrome c oxidase subunit viib	COX7B	0.0447	-1.14
Transmembrane protein 26	TMEM26	0.0117	-1.14
DEAD (Asp-Glu-Ala-Asp) box polypeptide 60	DDX60	0.0034	-1.14
Chromosome X open reading frame 58	CXorf58	0.0037	-1.14
Tetratricopeptide repeat domain 23-like	TTC23L	0.0209	-1.14
Transmembrane protein 108	TMEM108	0.0299	-1.14
Olfactory receptor, family 52, subfamily H, member 1	OR52H1	0.0321	-1.14
GA binding protein transcription factor alpha subunit	GABPA	0.0130	-1.14
Kallikrein related peptidase 12	KLK12	0.0412	-1.14
PHD finger protein 8	PHF8	0.0279	-1.14
Zinc finger and BTB domain containing 5	ZBTB5	0.0113	-1.14
Regulator of G-protein signaling 17	RGS17	0.0018	-1.14
Protein tyrosine phosphatase, non-receptor type 22 (lymphoid)	PTPN22	0.0173	-1.14
Chromosome 16 open reading	C16orf87	0.0035	-1.14

frame 87			
Olfactory receptor, family 11, subfamily H, member 12	OR11H12	0.0088	-1.14
Bromodomain adjacent to zinc finger domain 2B	BAZ2B	0.0287	-1.14
Solute carrier family 35 (GDP-fucose transporter), member C2	SLC35C2	0.0310	-1.14
Chromosome 8 open reading frame 89	C8orf89	0.0098	-1.15
Chromosome 22 open reading frame 31	C22orf31	0.0229	-1.15
BTG family, member 3	BTG3	0.0077	-1.15
Protocadherin beta 11	PCDHB11	0.0255	-1.15
DDB1 and CUL4 associated factor 10	DCAF10	0.0293	-1.15
Tumor protein p63 regulated 1	TPRG1	0.0457	-1.15
PDS5 cohesin associated factor B	PDS5B	0.0303	-1.15
Centriolin	CNTRL	0.0095	-1.15
Glutamyl-prolyl-trna synthetase	EPRS	0.0241	-1.15
Small ubiquitin-like modifier 4	SUMO4	0.0166	-1.15
Poliovirus receptor-related 3	PVRL3	0.0065	-1.15
Protein tyrosine phosphatase, non-receptor type 9	PTPN9	0.0465	-1.15
Zinc finger protein 319	ZNF319	0.0438	-1.15
ADAM metallopeptidase with thrombospondin type 1 motif 13	ADAMTS13	0.0418	-1.15
KIAA1143 [Source:HGNC Symbol;Acc:HGNC:29198]	KIAA1143	0.0110	-1.15
Dolichyldiphosphatase 1	DOLPP1	0.0337	-1.15
Adhesion G protein-coupled receptor D1	ADGRD1	0.0469	-1.15
Potassium channel, voltage gated Shal related subfamily D, member	KCND3	0.0452	-1.15
Lipocalin 12	LCN12	0.0328	-1.15
Rho GDP dissociation inhibitor (GDI) beta	ARHGDIB	0.0346	-1.15
Chromosome 3 open reading frame 22	C3orf22	0.0460	-1.15
ELOVL fatty acid elongase 7	ELOVL7	0.0354	-1.15
Core 1 synthase, glycoprotein-N-acetylgalactosamine 3-beta-gala	C1GALT1	0.0034	-1.15
Growth hormone regulated TBC protein 1	GRTP1	0.0347	-1.15
DEAD (Asp-Glu-Ala-Asp) box polypeptide 18	DDX18	0.0021	-1.15
Transcription factor EC	TFEC	0.0185	-1.15
Ring finger protein 5, E3 ubiquitin protein ligase	RNF5	0.0103	-1.15

Sp3 transcription factor	SP3	0.0406	-1.15
Memczak2013 ALT_ACCEPTOR, ALT_DONOR, coding, INTERNAL, int	YWHAG	0.0439	-1.15
Delta	DNER	0.0307	-1.15
Frizzled class receptor 3	FZD3	0.0148	-1.15
3-hydroxy-3-methylglutaryl-coa reductase	HMGCR	0.0150	-1.15
Deleted in azoospermia-like	DAZL	0.0365	-1.15
Thioredoxin	TXN	0.0192	-1.15
Itchy E3 ubiquitin protein ligase	ITCH	0.0239	-1.15
Neurobeachin like 2	NBEAL2	0.0433	-1.15
Diacylglycerol kinase, zeta	DGKZ	0.0199	-1.15
Glucosaminyl (N-acetyl) transferase 4, core 2	GCNT4	0.0308	-1.15
Beta-transducin repeat containing E3 ubiquitin protein ligase	BTRC	0.0072	-1.15
BEN domain containing 2	BEND2	0.0072	-1.15
Proline rich 4 (lacrimal)	PRR4	0.0031	-1.15
Histidine triad nucleotide binding protein 2	HINT2	0.0116	-1.15
Synaptonemal complex protein 3	SYCP3	0.0086	-1.15
Transient receptor potential cation channel, subfamily M, member	TRPM5	0.0069	-1.15
Autophagy related 16-like 1	ATG16L1	0.0277	-1.15
Bromodomain adjacent to zinc finger domain 1A	BAZ1A	0.0465	-1.15
Chromosome 2 open reading frame 88	C2orf88	0.0402	-1.15
Adenosylhomocysteinase-like 2	AHCYL2	0.0498	-1.15
Secreted frizzled-related protein 4	SFRP4	0.0170	-1.15
Dicer 1, ribonuclease type III	DICER1	0.0071	-1.15
Synaptonemal complex protein 1	SYCP1	0.0100	-1.15
Glycerophosphodiester phosphodiesterase 1	GDE1	0.0381	-1.15
T-box 18	TBX18	0.0207	-1.15
Acyl-coa thioesterase 13	ACOT13	0.0246	-1.15
ATP	AGTPBP1	0.0044	-1.15
V-rel avian reticuloendotheliosis viral oncogene homolog	REL	0.0227	-1.15
TBC1 domain family, member 32	TBC1D32	0.0066	-1.15
Ankyrin repeat and zinc finger domain containing 1	ANKZF1	0.0344	-1.15
Transmembrane protein 136	TMEM136	0.0191	-1.15
5,3-nucleotidase, mitochondrial	NT5M	0.0231	-1.15
CDC42 effector protein (Rho gtpase binding) 5	CDC42EP5	0.0067	-1.15
Butyrophilin, subfamily 1, member	BTN1A1	0.0198	-1.15

A1

Fc receptor, iga, igm, high affinity	FCAMR	0.0356	-1.15
KCTD21 antisense RNA 1	KCTD21-AS1	0.0237	-1.15
Defensin, beta 136	DEFB136	0.0155	-1.15
Major facilitator superfamily domain containing 12	MFSD12	0.0349	-1.15
MAX dimerization protein 4	MXD4	0.0237	-1.15
Neuropeptide Y receptor Y1	NPY1R	0.0170	-1.15
Small nuclear ribonucleoprotein D2 pseudogene 2	SNRPD2P2	0.0035	-1.15
TAR DNA binding protein	TARDBP	0.0034	-1.15
Zinc finger, DHHC-type containing 2	ZDHHC2	0.0460	-1.15
Transcript Identified by aceview, Entrez Gene ID(s) 238	ALK	0.0067	-1.15
Jeck2013 ALT_ACCEPTOR, ALT_DONOR, coding, INTERNAL, intron	SMYD3	0.0065	-1.15
Olfactory receptor, family 5, subfamily AU, member 1	OR5AU1	0.0344	-1.15
Dmx-like 2	DMXL2	0.0045	-1.15
Zinc finger protein 584	ZNF584	0.0226	-1.15
Tau tubulin kinase 2	TTBK2	0.0416	-1.15
HSPA (heat shock 70kda) binding protein, cytoplasmic cochaper	HSPBP1	0.0383	-1.15
FK506 binding protein 1B	FKBP1B	0.0235	-1.16
Nuclear factor, erythroid 2	NFE2	0.0329	-1.16
Nucleolar protein 4-like	NOL4L	0.0142	-1.16
Cytokine induced apoptosis inhibitor 1	CIAPIN1	0.0463	-1.16
Memczak2013 ALT_ACCEPTOR, ALT_DONOR, coding, INTERNAL, in	CAMKMT	0.0333	-1.16
Protein kinase, camp-dependent, regulatory, type I, alpha	PRKAR1A	0.0220	-1.16
Glutamate receptor, ionotropic, AMPA 1	GRIA1	0.0044	-1.16
Homer scaffolding protein 2	HOMER2	0.0425	-1.16
Rho gtpase activating protein 18	ARHGAP18	0.0211	-1.16
Mastermind-like domain containing 1	MAMLD1	0.0400	-1.16
COP9 signalosome subunit 8	COPS8	0.0411	-1.16
PTPRF interacting protein, binding protein 2 (liprin beta 2)	PPFIBP2	0.0248	-1.16
Oxysterol binding protein-like 11	OSBPL11	0.0310	-1.16
Lanc lantibiotic synthetase component C-like 1 (bacterial)	LANCL1	0.0038	-1.16
PAP associated domain containing 5	PAPD5	0.0276	-1.16
Lysine (K)-specific methyltransferase	KMT5A	0.0114	-1.16

5A			
Olfactory receptor, family 5, subfamily D, member 18	OR5D18	0.0004	-1.16
Chromosome 1 open reading frame 35	C1orf35	0.0226	-1.16
Olfactory receptor, family 10, subfamily R, member 2	OR10R2	0.0410	-1.16
Nuclear pore complex interacting protein family, member B11	NPIPB11	0.0124	-1.16
Sorting nexin 5	SNX5	0.0206	-1.16
WD repeat domain 75	WDR75	0.0115	-1.16
Putative uncharacterized protein FLJ37770-like	LOC100506127	0.0207	-1.16
Family with sequence similarity 81, member A	FAM81A	0.0019	-1.16
Calcium	CAMK2D	0.0352	-1.16
Syntrophin, gamma 1	SNTG1	0.0337	-1.16
NYN domain and retroviral integrase containing	NYNRIN	0.0040	-1.16
Olfactory receptor, family 10, subfamily AD, member 1	OR10AD1	0.0279	-1.16
Glutamate receptor, ionotropic, N-methyl D-aspartate 2B	GRIN2B	0.0452	-1.16
Ethylmalonic encephalopathy 1	ETHE1	0.0312	-1.16
Chloride channel, voltage-sensitive 3	CLCN3	0.0447	-1.16
F-box protein 32	FBXO32	0.0321	-1.16
Hemopexin	HPX	0.0190	-1.16
Keratin 32, type I	KRT32	0.0324	-1.16
Hydroxyacid oxidase (glycolate oxidase) 1	HAO1	0.0261	-1.16
Non-POU domain containing, octamer-binding	NONO	0.0071	-1.16
Transcript Identified by aceview, Entrez Gene ID(s	GRIN2A	0.0199	-1.16
Dual specificity phosphatase 15	DUSP15	0.0174	-1.16
Fibroblast growth factor 12	FGF12	0.0384	-1.16
Lamin B1	LMNB1	0.0238	-1.16
Proteasome activator subunit 3	PSME3	0.0461	-1.16
Nucleosome assembly protein 1-like 6	NAP1L6	0.0187	-1.16
Nucleophosmin	NPM3	0.0315	-1.16
ADP-ribosylation factor like gtpase 5C	ARL5C	0.0241	-1.16
Histone cluster 1, h2bi	HIST1H2BI	0.0154	-1.16
Zinc finger and SCAN domain containing 4	ZSCAN4	0.0200	-1.16

Tripartite motif containing 55	TRIM55	0.0344	-1.16
Synovial sarcoma, X breakpoint 6 (pseudogene)	SSX6	0.0218	-1.16
Transmembrane protein 192	TMEM192	0.0083	-1.16
RAD51 paralog C	RAD51C	0.0281	-1.16
DENN	DENND2C	0.0446	-1.16
Secretogranin III	SCG3	0.0100	-1.16
Pantothenate kinase 1	PANK1	0.0427	-1.16
Exportin 1	XPO1	0.0060	-1.16
Zinc finger protein 197	ZNF197	0.0207	-1.16
Zinc finger protein 37A	ZNF37A	0.0219	-1.16
Zinc finger protein 571	ZNF571	0.0120	-1.16
Serpin peptidase inhibitor, clade A (alpha-1 antiproteinase, a	SERPINA6	0.0208	-1.16
Sulfotransferase family 1E member 1	SULT1E1	0.0207	-1.16
DEAD (Asp-Glu-Ala-Asp) box polypeptide 27	DDX27	0.0096	-1.16
Phosphoglucomutase 2-like 1	PGM2L1	0.0143	-1.16
Acidic nuclear phosphoprotein 32 family member D	ANP32D	0.0120	-1.16
Protein tyrosine phosphatase, mitochondrial 1	PTPMT1	0.0191	-1.16
Cerebellin 4 precursor	CBLN4	0.0077	-1.16
ARP1 actin-related protein 1 homolog B, centractin beta (yeast)	ACTR1B	0.0280	-1.16
Sperm mitochondria-associated cysteine-rich protein	SMCP	0.0091	-1.16
FYVE, rhogef and PH domain containing 6	FGD6	0.0028	-1.16
R3H domain containing 2	R3HDM2	0.0269	-1.17
Leucine rich repeat containing 59	LRRC59	0.0213	-1.17
Signal-regulatory protein gamma	SIRPG	0.0232	-1.17
Phosphofructokinase, platelet	PFKP	0.0077	-1.17
Lectin, galactoside-binding-like	LGALSL	0.0435	-1.17
FYN binding protein	FYB	0.0328	-1.17
Zinc finger and BTB domain containing 14	ZBTB14	0.0119	-1.17
SIX homeobox 4	SIX4	0.0138	-1.17
ZBED6 C-terminal like	ZBED6CL	0.0232	-1.17
Protein phosphatase 1, regulatory subunit 17	PPP1R17	0.0421	-1.17
Tryptase delta 1	TPSD1	0.0278	-1.17
Ankyrin repeat domain 1 (cardiac muscle)	ANKRD1	0.0192	-1.17
PTGES3L-AARSD1 readthrough	PTGES3L-AARSD1	0.0337	-1.17

Ras-related GTP binding B	RRAGB	0.0180	-1.17
Protocadherin beta 15	PCDHB15	0.0252	-1.17
Mbt domain containing 1	MBTD1	0.0120	-1.17
TATA box binding protein associated factor 7 like	TAF7L	0.0431	-1.17
Meiosis specific with OB domains	MEIOB	0.0105	-1.17
Praja ring finger 2, E3 ubiquitin protein ligase	PJA2	0.0046	-1.17
Transmembrane protein 114	TMEM114	0.0276	-1.17
Lymphocyte-specific protein 1 pseudogene 3	LSP1P3	0.0311	-1.17
Lipocalin 8	LCN8	0.0363	-1.17
Transcript Identified by aceview, Entrez Gene ID(s) 6	TBCD	0.0268	-1.17
Patched domain containing 3	PTCHD3	0.0253	-1.17
Mediator complex subunit 23	MED23	0.0232	-1.17
Leucine rich repeat containing 8 family, member C	LRRC8C	0.0413	-1.17
Kelch-like family member 18	KLHL18	0.0206	-1.17
WAPL cohesin release factor	WAPL	0.0180	-1.17
Ubiquitin specific peptidase 32 pseudogene 2	USP32P2	0.0283	-1.17
Transmembrane protease, serine 11A	TMPRSS11A	0.0064	-1.17
BCL2-related protein A1	BCL2A1	0.0185	-1.17
Peptidyl arginine deiminase, type VI	PADI6	0.0236	-1.17
S100 calcium binding protein A12	S100A12	0.0168	-1.17
Exosome component 6	EXOSC6	0.0338	-1.17
Integrator complex subunit 3	INTS3	0.0194	-1.17
Factor interacting with PAPOLA and CPSF1	FIP1L1	0.0140	-1.17
RNA binding motif protein 41	RBM41	0.0036	-1.17
Chromosome 1 open reading frame 52	C1orf52	0.0201	-1.17
Neuroblastoma breakpoint family, member 14	NBPF14	0.0181	-1.17
Chromosome 4 open reading frame 32	C4orf32	0.0053	-1.17
TGFB-induced factor homeobox 1	TGIF1	0.0222	-1.17
Memczak2013 ANTISENSE, CDS, coding, INTERNAL best transcr	ATP2A3	0.0359	-1.17
Defensin, beta 108B	DEFB108B	0.0236	-1.17
Protein phosphatase 1, regulatory subunit 16A	PPP1R16A	0.0230	-1.17
POU class 5 homeobox 1B	POU5F1B	0.0437	-1.17
Adhesion molecule, interacts with CXADR antigen 1	AMICA1	0.0475	-1.17

Transcript Identified by aceview, Entrez Gene ID(	ARPP21	0.0195	-1.17
RAB guanine nucleotide exchange factor (GEF) 1	RABGEF1	0.0228	-1.17
Synapsin I	SYN1	0.0304	-1.17
TAO kinase 1	TAOK1	0.0099	-1.17
Polymerase (DNA directed), alpha 2, accessory subunit	POLA2	0.0104	-1.17
Nurim (nuclear envelope membrane protein)	NRM	0.0140	-1.17
Sterile alpha motif domain containing 8	SAMD8	0.0317	-1.17
Growth hormone releasing hormone	GHRH	0.0402	-1.17
Nucleolar protein 4	NOL4	0.0068	-1.17
Protein tyrosine phosphatase, receptor type, f polypeptide (PTPR	PPFIA3	0.0230	-1.17
SH2 domain containing 7	SH2D7	0.0188	-1.18
Thiopurine S-methyltransferase	TPMT	0.0186	-1.18
Myosin light chain 10	MYL10	0.0119	-1.18
Transcript Identified by aceview, Entrez Gene ID(s) 55	EPN3	0.0431	-1.18
Dedicator of cytokinesis 4	DOCK4	0.0021	-1.18
RAB34, member RAS oncogene family	RAB34	0.0400	-1.18
Hypermethylated in cancer 2	HIC2	0.0073	-1.18
Transmembrane protein 170B	TMEM170B	0.0283	-1.18
Mediator of DNA-damage checkpoint 1	MDC1	0.0393	-1.18
Transient receptor potential cation channel, subfamily C, memb	TRPC7	0.0108	-1.18
Dual specificity tyrosine-(Y)-phosphorylation regulated ki	DYRK1B	0.0496	-1.18
Aspartylglucosaminidase	AGA	0.0115	-1.18
Spindle apparatus coiled-coil protein 1	SPDL1	0.0081	-1.18
SRY box 6	SOX6	0.0293	-1.18
Phosphoribosyl pyrophosphate synthetase 1	PRPS1	0.0046	-1.18
BUD13 homolog	BUD13	0.0144	-1.18
Forkhead box A1	FOXA1	0.0292	-1.18
Radixin	RDX	0.0178	-1.18
Translocase of outer mitochondrial membrane 70 homolog A (S. Ce	TOMM70A	0.0109	-1.18
Transmembrane protein 213	TMEM213	0.0394	-1.18
Solute carrier family 4 (sodium bicarbonate cotransporter), m	SLC4A4	0.0189	-1.18

Chromosome 2 open reading frame 49	C2orf49	0.0094	-1.18
Frequently rearranged in advanced T-cell lymphomas 1	FRAT1	0.0355	-1.18
Collagen, type XVII, alpha 1	COL17A1	0.0090	-1.18
Muscular LMNA-interacting protein	MLIP	0.0186	-1.18
Superoxide dismutase 3, extracellular	SOD3	0.0370	-1.18
Olfactory receptor, family 2, subfamily T, member 35	OR2T35	0.0157	-1.18
TRAF-type zinc finger domain containing 1	TRAFD1	0.0127	-1.18
FXYD domain containing ion transport regulator 5	FXYD5	0.0436	-1.18
Synaptoporin	SYNPR	0.0164	-1.18
Phosphatidylinositol glycan anchor biosynthesis class G	PIGG	0.0200	-1.18
Family with sequence similarity 157, member A	FAM157A	0.0303	-1.18
Forkhead box J3	FOXJ3	0.0055	-1.18
Coiled-coil domain containing 138	CCDC138	0.0133	-1.18
Zinc finger protein 526	ZNF526	0.0050	-1.18
Chromogranin B	CHGB	0.0244	-1.18
S-antigen; retina and pineal gland (arrestin)	SAG	0.0225	-1.18
Growth arrest-specific 2	GAS2	0.0157	-1.18
Sema domain, transmembrane domain (TM), and cytoplasmic domai	SEMA6D	0.0316	-1.18
Peptidylprolyl isomerase B (cyclophilin B)	PPIB	0.0083	-1.18
G protein-coupled receptor 155	GPR155	0.0053	-1.18
Heparanase 2 (inactive)	HPSE2	0.0117	-1.18
LDL receptor related protein 6	LRP6	0.0042	-1.18
PDZ and LIM domain 4	PDLIM4	0.0196	-1.18
Adaptor-related protein complex 3, sigma 2 subunit	AP3S2	0.0366	-1.18
ATP	AGBL4	0.0076	-1.18
Structural maintenance of chromosomes 5	SMC5	0.0072	-1.19
Prostate and testis expressed 4	PATE4	0.0445	-1.19
Histone cluster 1, H1d	HIST1H1D	0.0276	-1.19
Calcium channel, voltage-dependent, beta 1 subunit	CACNB1	0.0144	-1.19
Protein tyrosine phosphatase, receptor type, T	PTPRT	0.0114	-1.19
Methylenetetrahydrofolate dehydrogenase (NADP+ dependent)	MTHFD2	0.0014	-1.19

2, met			
Torsin family 1, member A (torsin A)	TOR1A	0.0084	-1.19
Transcriptional regulating factor 1	TRERF1	0.0228	-1.19
Myeloid	MLLT1	0.0409	-1.19
Ral gtpase activating protein, alpha subunit 2 (catalytic)	RALGAPA2	0.0140	-1.19
Immunoglobulin superfamily, member 8	IGSF8	0.0091	-1.19
Ubiquitin-conjugating enzyme E2D 4 (putative)	UBE2D4	0.0052	-1.19
Family with sequence similarity 185, member A	FAM185A	0.0100	-1.19
Kinesin family member 22	KIF22	0.0334	-1.19
Mediator complex subunit 20	MED20	0.0313	-1.19
Sphingomyelin phosphodiesterase, acid-like 3A	SMPDL3A	0.0083	-1.19
Chromosome X open reading frame 57	CXorf57	0.0134	-1.19
Ubiquitin specific peptidase 9, Y-linked	USP9Y	0.0331	-1.19
SOGA family member 3	SOGA3	0.0180	-1.19
Coiled-coil domain containing 126	CCDC126	0.0136	-1.19
Peptidase inhibitor 15	PI15	0.0134	-1.19
Memczak2013 ALT_ACCEPTOR, ALT_DONOR, coding, INTERNAL, int	CCND3	0.0294	-1.19
Enhancer of yellow 2 homolog (Drosophila)	ENY2	0.0070	-1.19
Cilia and flagella associated protein 47	CFAP47	0.0100	-1.19
SPATA31 subfamily D, member 3	SPATA31D3	0.0053	-1.19
Purinergic receptor P2Y, G-protein coupled, 13	P2RY13	0.0403	-1.19
Polymerase (RNA) I polypeptide B	POLR1B	0.0060	-1.19
Neurotrophin 3	NTF3	0.0273	-1.19
Nuclear transcription factor, X-box binding 1	NFX1	0.0402	-1.19
FGR proto-oncogene, Src family tyrosine kinase	FGR	0.0064	-1.19
Dishevelled associated activator of morphogenesis 1	DAAM1	0.0091	-1.19
Nitric oxide synthase trafficking	NOSTRIN	0.0022	-1.19
Transcript Identified by aceview, Entrez Gene ID(s)	THSD4	0.0235	-1.19
Doublecortin domain containing 2B	DCDC2B	0.0432	-1.19
G protein-coupled receptor 150	GPR150	0.0175	-1.19
Bromodomain, testis-specific	BRDT	0.0008	-1.19
Uncharacterized LOC101929319	LOC101929319	0.0089	-1.19

Adenylate cyclase 4	ADCY4	0.0141	-1.19
ADNP homeobox 2	ADNP2	0.0105	-1.19
Transmembrane protein 67	TMEM67	0.0037	-1.19
Olfactomedin like 3	OLFML3	0.0137	-1.19
N-acetylneuraminate pyruvate lyase (dihydrodipicolinate synthase	NPL	0.0068	-1.19
Olfactory receptor, family 7, subfamily A, member 10	OR7A10	0.0072	-1.19
Keratin associated protein 3-1	KRTAP3-1	0.0496	-1.19
Discoidin domain receptor tyrosine kinase 1	DDR1	0.0175	-1.19
Thrombopoietin	THPO	0.0098	-1.19
Coiled-coil domain containing 175	CCDC175	0.0070	-1.19
Acetyl-coa acyltransferase 1	ACAA1	0.0062	-1.19
Coiled-coil domain containing 34	CCDC34	0.0279	-1.20
G protein-coupled receptor 31	GPR31	0.0463	-1.20
PRAME family member 6 [Source:HGNC Symbol;Acc:HGNC:30583]	PRAMEF6	0.0320	-1.20
PRAME family member 6 [Source:HGNC Symbol;Acc:HGNC:30583]	PRAMEF6	0.0320	-1.20
CD109 molecule	CD109	0.0034	-1.20
Synergin, gamma	SYNRG	0.0070	-1.20
Stearoyl-coa desaturase 5	SCD5	0.0163	-1.20
Trafficking protein particle complex 2-like	TRAPPC2L	0.0317	-1.20
Regulator of microtubule dynamics 3	RMDN3	0.0099	-1.20
Ankyrin repeat domain 20 family, member A1	ANKRD20A1	0.0234	-1.20
SHANK-associated RH domain interactor	SHARPIN	0.0271	-1.20
KIAA1644	KIAA1644	0.0179	-1.20
Prostate and testis expressed 1	PATE1	0.0056	-1.20
Myelin protein zero-like 2	MPZL2	0.0171	-1.20
Collagen, type XX, alpha 1	COL20A1	0.0056	-1.20
Neuropeptide FF receptor 1	NPFFR1	0.0052	-1.20
Coiled-coil domain containing 129	CCDC129	0.0013	-1.20
RAD21 cohesin complex component like 1	RAD21L1	0.0074	-1.20
Rho guanine nucleotide exchange factor (GEF) 12	ARHGEF12	0.0133	-1.20
Kelch-like family member 42	KLHL42	0.0124	-1.20
Myomesin 3	MYOM3	0.0161	-1.20
Family with sequence similarity 124 member B	FAM124B	0.0113	-1.20

Plexin D1	PLXND1	0.0305	-1.20
Transcript Identified by aceview, Entrez Gene ID(s) 13	UBR3	0.0235	-1.20
ADP-ribosylation factor like gtpase 5B	ARL5B	0.0205	-1.20
TBC1 domain family, member 13	TBC1D13	0.0460	-1.20
Matrix metallopeptidase 19	MMP19	0.0092	-1.20
Essential meiotic structure-specific endonuclease 1	EME1	0.0120	-1.20
Zinc finger protein 627	ZNF627	0.0274	-1.20
Transcript Identified by aceview, Entrez Gene ID(s) 65	HLTF	0.0312	-1.20
5-phosphohydroxy-L-lysine phospho-lyase	PHYKPL	0.0457	-1.20
Leucine rich repeat containing 39	LRRC39	0.0004	-1.20
Slingshot protein phosphatase 2	SSH2	0.0163	-1.20
G protein-coupled receptor 42 (gene	GPR42	0.0339	-1.20
Synaptosome associated protein 29kda	SNAP29	0.0317	-1.20
Chromosome 2 open reading frame 80	C2orf80	0.0018	-1.20
Transcript Identified by aceview, Entrez Gene ID(s)	TRABD	0.0112	-1.20
Patatin-like phospholipase domain containing 2	PNPLA2	0.0265	-1.20
Transcript Identified by aceview, Entrez Gene ID(s) 41	CD46	0.0061	-1.20
RBM14-RBM4 readthrough	RBM14-RBM4	0.0244	-1.20
Long intergenic non-protein coding RNA 337	LINC00337	0.0065	-1.20
Dnaj (Hsp40) homolog, subfamily B, member 12	DNAJB12	0.0043	-1.20
G-rich RNA sequence binding factor 1	GRSF1	0.0019	-1.20
Chromosome 9 open reading frame 106	C9orf106	0.0017	-1.20
Olfactory receptor, family 5, subfamily AC, member 2	OR5AC2	0.0053	-1.20
Transcript Identified by aceview, Entrez Gene ID(s)	WDR26	0.0030	-1.20
Chromosome 7 open reading frame 60	C7orf60	0.0032	-1.20
Coagulation factor XI	F11	0.0337	-1.20
Zinc finger with KRAB and SCAN domains 3	ZKSCAN3	0.0019	-1.20
Splicing factor proline	SFPQ	0.0073	-1.20

F-box protein 44	FBXO44	0.0119	-1.20
IZUMO family member 3	IZUMO3	0.0034	-1.20
Zinc finger protein 780A	ZNF780A	0.0004	-1.20
Crystallin beta A4	CRYBA4	0.0377	-1.21
Fatty acid desaturase 3	FADS3	0.0343	-1.21
Transmembrane protein 159	TMEM159	0.0031	-1.21
Dorsal inhibitory axon guidance protein	DRAXIN	0.0055	-1.21
Nuclear pore complex interacting protein family, member B8	NPIPB8	0.0103	-1.21
Phosphatase and actin regulator 3	PHACTR3	0.0141	-1.21
Polymerase (DNA directed), eta	POLH	0.0144	-1.21
Golgi glycoprotein 1	GLG1	0.0212	-1.21
5,10-methenyltetrahydrofolate synthetase (5-formyltetrahydrofo	MTHFS	0.0112	-1.21
Sema domain, immunoglobulin domain (Ig), transmembrane domain (T	SEMA4B	0.0017	-1.21
TAF1 RNA polymerase II, TATA box binding protein (TBP)-associat	TAF1	0.0030	-1.21
Dual specificity tyrosine-(Y)-phosphorylation regulated kinase	DYRK4	0.0168	-1.21
Cerberus 1, DAN family BMP antagonist	CER1	0.0077	-1.21
Eukaryotic translation initiation factor 2 alpha kinase 4	EIF2AK4	0.0082	-1.21
Angiopoietin 1	ANGPT1	0.0012	-1.21
Signal recognition particle receptor (docking protein)	SRPR	0.0118	-1.21
Leucyl-trna synthetase 2, mitochondrial	LARS2	0.0099	-1.21
IQ motif containing C	IQCC	0.0237	-1.21
Four and a half LIM domains 2	FHL2	0.0152	-1.21
Palmdelphin	PALMD	0.0187	-1.21
Olfactory receptor, family 3, subfamily A, member 3	OR3A3	0.0435	-1.21
Ribosomal protein S6 kinase, 90kda, polypeptide 3	RPS6KA3	0.0003	-1.21
Zinc finger protein 80	ZNF80	0.0155	-1.21
Ubiquitin specific peptidase 2	USP2	0.0095	-1.21
Memczak2013 ALT_ACCEPTOR, ALT_DONOR, coding, INTERNAL, int	CNPY3	0.0461	-1.21
Complement factor I	CFI	0.0091	-1.21
KRAB box domain containing 1	KRBOX1	0.0009	-1.21
UXT antisense RNA 1	UXT-AS1	0.0114	-1.21
Protein regulator of cytokinesis 1	PRC1	0.0299	-1.22
RNA binding motif protein 26	RBM26	0.0014	-1.22

Gap junction protein alpha 1	GJA1	0.0169	-1.22
Nuclear factor of activated T-cells, cytoplasmic, calcineurin	NFATC4	0.0027	-1.22
KIAA1211	KIAA1211	0.0030	-1.22
DNA meiotic recombinase 1	DMC1	0.0006	-1.22
Surfeit 1	SURF1	0.0268	-1.22
Motor neuron and pancreas homeobox 1	MNX1	0.0304	-1.22
Myosin, heavy chain 2, skeletal muscle, adult	MYH2	0.0005	-1.22
Protein-L-isoaspartate (D-aspartate) O-methyltransferase	PCMT1	0.0035	-1.22
Zinc finger protein 337	ZNF337	0.0367	-1.22
Solute carrier family 7, member 6 opposite strand	SLC7A6OS	0.0085	-1.22
PDZ domain containing 11	PDZD11	0.0170	-1.22
Homeobox B7	HOXB7	0.0025	-1.22
Troponin T type 2 (cardiac)	TNNT2	0.0034	-1.22
PRAME family member 6 [Source:HGNC Symbol;Acc:HGNC:30583]	PRAMEF6	0.0240	-1.22
Cytochrome P450, family 4, subfamily B, polypeptide 1	CYP4B1	0.0009	-1.22
Chromosome 1 open reading frame 131	C1orf131	0.0016	-1.23
Hemicentin 1	HMCN1	0.0121	-1.23
Vertebrae development associated	VRTN	0.0130	-1.23
Transcript Identified by aceview, Entrez Gene ID(s)	MTMR6	0.0220	-1.23
Memczak2013 ANTISENSE, CDS, coding, INTERNAL best transcri	MYADM	0.0387	-1.23
Zhang2013 ALT_ACCEPTOR, ALT_DONOR, coding, INTERNAL, intron	NINL	0.0140	-1.23
Transcript Identified by aceview, Entrez Gene ID(s)	ALG13	0.0117	-1.23
Cartilage intermediate layer protein, nucleotide pyrophosphohydrol	CILP	0.0027	-1.23
Golgi-associated, gamma adaptin ear containing, ARF binding protei	GGA2	0.0035	-1.23
Mitochondrial ribosomal protein L54	MRPL54	0.0063	-1.23
Interleukin 16	IL16	0.0080	-1.24
Upregulator of cell proliferation	URGCP	0.0050	-1.24
Jeck2013 ANTISENSE, coding, INTERNAL, intronic best transc	KIFC3	0.0081	-1.24
Myoregulin	MRLN	0.0046	-1.24

Gastrin-releasing peptide	GRP	0.0013	-1.24
RAS (RAD and GEM)-like GTP-binding 1	REM1	0.0072	-1.24
Hypoxia up-regulated 1	HYOU1	0.0058	-1.24
Monoacylglycerol O-acyltransferase 2	MOGAT2	0.0046	-1.24
Retinoic acid receptor responder (tazarotene induced) 3	RARRES3	0.0095	-1.24
Potassium channel, two pore domain subfamily K, member 2	KCNK2	0.0106	-1.24
Sorting nexin 1	SNX1	0.0064	-1.24
Chromosome 12 open reading frame 60	C12orf60	0.0022	-1.24
RNA binding protein with multiple splicing 2	RBPMS2	0.0090	-1.24
ZNF559-ZNF177 readthrough	ZNF559-ZNF177	0.0008	-1.24
Cerebellar degeneration related protein 2	CDR2	0.0125	-1.24
Limbic system-associated membrane protein	LSAMP	0.0124	-1.25
Immunoglobin superfamily, member 21	IGSF21	0.0096	-1.25
IQ motif containing gtpase activating protein 2	IQGAP2	0.0083	-1.25
Ubiquitin specific peptidase 4 (proto-oncogene)	USP4	0.0127	-1.25
Transient receptor potential cation channel, subfamily V, member	TRPV6	0.0164	-1.25
Phosphatase and actin regulator 1	PHACTR1	0.0054	-1.25
Proteasome 26S subunit, non-atpase 14	PSMD14	0.0294	-1.25
Memczak2013 ALT_ACCEPTOR, ALT_DONOR, coding, INTERNAL, intr	MSI2	0.0276	-1.25
Disrupted in renal carcinoma 3	DIRC3	0.0164	-1.25
Chemokine (C-C motif) ligand 27	CCL27	0.0346	-1.26
Gap junction protein gamma 2	GJC2	0.0029	-1.26
Uncharacterized LOC100505478	LOC100505478	0.0132	-1.26
Solute carrier family 22 (organic anion transporter), member	SLC22A8	0.0104	-1.26
Memczak2013 ALT_ACCEPTOR, ALT_DONOR, INTERNAL, intronic, nc	GAS8	0.0113	-1.26
Chromosome 11 open reading frame 87	C11orf87	0.0002	-1.26
Transcript Identified by aceview, Entrez Gene ID(s)	NCOA7	0.0009	-1.26
CD79b molecule, immunoglobulin-associated beta	CD79B	0.0053	-1.26

CXADR-like membrane protein	CLMP	0.0390	-1.26
SAFB-like, transcription modulator	SLTM	0.0228	-1.26
TSPY-like 6	TSPYL6	0.0051	-1.27
Fibrillarin	FBL	0.0005	-1.27
Transcript Identified by aceview, Entrez Gene I	SLCO1A2	0.0002	-1.27
Transcript Identified by aceview, Entrez Gene ID(	TRIP12	0.0033	-1.27
Olfactory receptor, family 10, subfamily J, member 3	OR10J3	0.0012	-1.27
Component of oligomeric golgi complex 1	COG1	0.0253	-1.27
Lectin, galactoside-binding, soluble, 16	LGALS16	0.0001	-1.27
Memczak2013 ANTISENSE, CDS, coding, INTERNAL, intronic b	SLC10A1	0.0193	-1.28
Family with sequence similarity 83, member G	FAM83G	0.0018	-1.28
ADP-ribosyltransferase 3	ART3	0.0017	-1.28
PR domain containing 6	PRDM6	0.0007	-1.28
Microtubule associated tumor suppressor 1	MTUS1	0.0020	-1.28
Neuroblastoma breakpoint family, member 7	NBPF7	0.0000	-1.28
RFT1 homolog	RFT1	0.0003	-1.29
Solute carrier family 9, subfamily A (NHE7, cation proton ant	SLC9A7	0.0001	-1.31
PRAME family member 5	PRAMEF5	0.0002	-1.31
Neuronal regeneration related protein	NREP	0.0005	-1.33
Transcript Identified by aceview, Entrez Gene ID(s) 34	FMN1	0.0036	-1.33
Memczak2013 ALT_ACCEPTOR, ALT_DONOR, coding, INTERNAL, in	PRPF19	0.0001	-1.34
Solute carrier family 31 (copper transporter), member 1	SLC31A1	0.0047	-1.34
Integrin beta 4	ITGB4	0.0000	-1.36
Mucin 12, cell surface associated	MUC12	0.0020	-1.38
Olfactory receptor, family 9, subfamily Q, member 2	OR9Q2	0.0000	-1.44
Neutrophil cytosolic factor 1	NCF1	0.0446	-1.59
